# CDC Program Evaluation Framework, 2024

**DOI:** 10.15585/mmwr.rr7306a1

**Published:** 2024-09-26

**Authors:** Daniel P. Kidder, Leslie A. Fierro, Elena Luna, Heather Salvaggio, Amanda McWhorter, Shelly-Ann Bowen, Rebecca Murphy-Hoefer, Sally Thigpen, Dayna Alexander, Theresa L. Armstead, Euna August, Destiny Bruce, Seseni Nu Clarke, Cassandra Davis, Amia Downes, Sarah Gill, L. Duane House, Michael Kerzner, Karen Kun, Karen Mumford, Leah Robin, Dara Schlueter, Michael Schooley, Eduardo Valverde, Linda Vo, Donjanea Williams, Kai Young, CDC Evaluation Framework Work Group, CDC Evaluation Framework Work Group Members, Anita Alston Jones, Bayo Arthur, Omoshalewa Bamkole, Diana Bartlett, Mohamed Bouras, Christopher J. Cappelli, Denise C. Carty, Jessica Crowell, Shivani Dama, Jina Dcruz, Dora Ducak, Tambra Dunams, Arlene Edwards, Oluwayomi Fabayo, Leah S. Fischer, Holly H. Fisher, Cassandra Martin Frazier, Sherese Bleechington Garrett, Danique Gigger, Danielle Gilliard Pella, Jeffrey M. Gordon, Bradford Greening, Jordan D. Helms, Sara Jacenko, Jill Kuhn, Nicole Kuiper, S. Rene Lavinghouze, Neranga Liyanaarachchige, Elizabeth Lummus, Brandy L. Peterson, Maddox, Angèle Marandet, Mariette Marano-Lee, Brittany Marshall, Elizabeth Martin, Caitlin McColloch, Susi McGhee, Carol Nixon, Lijing Ouyang, Jasmine R. Owens, Margaret Paek, Suchita Patel, Miriam Phields, Shubha Rao, Minda D. Reed, Michelle Roth, Maria Sanchez, Delight Satter, Arielle Shiver, Kat Sisler, Amrita Tailor, Affaud Tanon, Andrea Torres, Silvia M. Trigoso, Ann Ussery-Hall, Ijeoma Uzoezie, Nga Vuong, Maureen Wilce, Shaunta S. Wright, Monique Young

**Affiliations:** ^1^Office of Policy, Performance, and Evaluation, CDC, Atlanta, Georgia; ^2^Fierro Consulting, Inc., Thousand Oaks, California; ^3^Max Bell School of Public Policy, McGill University, Montreal, Quebec, Canada; ^4^Office of Readiness and Response, Division of State and Local Readiness, CDC, Atlanta, Georgia; ^5^Division of Diabetes Translation, National Center for Chronic Disease Prevention and Health Promotion, CDC, Atlanta, Georgia; ^6^Office on Smoking and Health, Center for Chronic Disease Prevention and Health Promotion, CDC, Atlanta, Georgia; ^7^Office of the Director, National Center for Injury Prevention and Control, CDC, Atlanta, Georgia; ^8^Office of Data Reporting and Evaluation, National Center for State, Tribal, Local and Territorial Infrastructure and Workforce, CDC, Atlanta, Georgia; ^9^Office of Health Equity, CDC, Atlanta, Georgia; ^10^Division of Global HIV and TB, Global Health Center, CDC, Atlanta, Georgia; ^11^Office of the Director, National Institute for Occupational Safety and Health, CDC, Atlanta, Georgia; ^12^Division of Environmental Health Science and Practice, National Center for Environmental Health, CDC, Atlanta, Georgia; ^13^Division of Population Health, National Center for Chronic Disease Prevention and Health Promotion, CDC, Atlanta, Georgia; ^14^Division of HIV Prevention, National Center for HIV, Viral Hepatitis, STD, and Tuberculosis Prevention, CDC, Atlanta, Georgia; ^15^Division of Jurisdictional Support, Public Health Infrastructure Center, CDC, Atlanta, Georgia; ^16^Division of Adolescent and School Health, National Center for Chronic Disease Prevention and Health Promotion, CDC, Atlanta, Georgia; ^17^Division of Cancer Prevention and Control, National Center for Chronic Disease Prevention and Health Promotion, CDC, Atlanta, Georgia; ^18^Division for Heart Disease and Stroke Prevention, National Center for Chronic Disease Prevention and Health Promotion, CDC, Atlanta, Georgia; ^19^Office of the Director, National Center for HIV, Viral Hepatitis, STD, and Tuberculosis Prevention, CDC, Atlanta, Georgia; ^20^Division of Overdose Prevention, National Center for Injury Prevention and Control, CDC, Atlanta, Georgia; National Institute for Occupational Safety and Health; CDC; Immunization Services Division; National Center for Immunization and Respiratory Diseases; CDC; Division of Workforce Development; National Center for State, Tribal, Local, and Territorial Public Health Infrastructure and Workforce; CDC; Office of Science; CDC; Office of the Director; National Center for Chronic Disease Prevention and Health Promotion,; CDC; Division of Global HIV & TB; Global Health Center; CDC; Office of Women's Health; CDC; Division of Violence Prevention; National Center for Injury Prevention and Control; CDC; Division of STD Prevention; National Center for HIV, Viral Hepatitis, STD, and Tuberculosis Prevention; CDC; Office of the Director; National Center for Immunization and Respiratory Diseases; CDC; Division of Violence Prevention; National Center for Injury Prevention and Control; CDC; Division of Emergency Operations; Office of Readiness and Response; CDC; Division of HIV Prevention; National Center for HIV, Viral Hepatitis, STD, and Tuberculosis Prevention; CDC; Division of Diabetes Translation; National Center for Chronic Disease Prevention and Health Promotion; CDC; Division of Infectious Disease Readiness and Innovation; National Center for Emerging and Zoonotic Infectious Diseases; CDC; Division of Viral Hepatitis; National Center for HIV, Viral Hepatitis, STD, and Tuberculosis Prevention; CDC; Division of Partnership Support; National Center for State, Tribal, Local, and Territorial Public Health Infrastructure and Workforce; CDC; Division of HIV Prevention; National Center for HIV, Viral Hepatitis, STD, and Tuberculosis Prevention; CDC; Division of Global Health Protection; Global Health Center; CDC; Office of the Director; National Center for Emerging and Zoonotic Infectious Diseases; CDC; Division of Workforce Development; Public Health Infrastructure Center; CDC; Division of Infectious Disease Readiness and Innovation; National Center for Emerging and Zoonotic Infectious Diseases; CDC; Division of Laboratory Systems, Center for Laboratory Systems and Responses; CDC; Global Immunization Division; Global Health Center; CDC; Office of Integration and Coordination; Global Health Center; CDC; Division of Population Health; National Center for Chronic Disease Prevention and Health Promotion; CDC; Division of Nutrition, Physical Activity, and Obesity; National Center for Chronic Disease Prevention and Health Promotion; CDC; Division of Global HIV and TB; Global Health Center; CDC; Division of Overdose Prevention; National Center for Injury Prevention and Control; CDC; Office on Smoking and Health; National Center for Chronic Disease Prevention and Promotion; CDC; Division of Global HIV and TB; Global Health Center; CDC; Division of HIV Prevention; National Center for HIV, Viral Hepatitis, STD, and Tuberculosis Prevention; CDC; Division of HIV Prevention; National Center for HIV, Viral Hepatitis, STD, and Tuberculosis Prevention; CDC; Office of Equal Employment Opportunity and Workplace Equity; CDC; Division of Workforce Development; National Center for State, Tribal, Local, and Territorial Public Health Infrastructure and Workforce; CDC; Division of Injury Prevention; National Center for Injury Prevention and Control; CDC; Spokane Mining Research Division; National Institute for Occupational Safety and Health; CDC; Division of Reproductive Health; National Center for Chronic Disease Prevention and Health Promotion; CDC; Division of Overdose Prevention; National Center for Injury Prevention and Control; CDC; Division of Healthcare Quality Promotion; National Center for Emerging and Zoonotic Infectious Diseases; CDC; Immunization Services Division; National Center for Immunization and Respiratory Diseases; CDC; Division of HIV Prevention; National Center for HIV, Viral Hepatitis, STD, and Tuberculosis Prevention; CDC; Division of HIV Prevention; Division of Viral Hepatitis; National Center for HIV, Viral Hepatitis, STD, and Tuberculosis Prevention; CDC; Division of Overdose Prevention; National Center for Injury Prevention and Control; CDC; Division of Foodborne, Waterborne, and Environmental Diseases; National Center for Emerging and Zoonotic Infectious Diseases; CDC; Division of Human Development and Disability; National Center on Birth Defects and Developmental Disabilities; CDC; Office of the Director; National Center for State, Tribal, Local, and Territorial Public Health Infrastructure and Workforce; CDC; Division of Nutrition, Physical Activity, and Obesity; National Center for Chronic Disease Prevention and Health Promotion; CDC; Division of Environmental Health Science and Practice; National Center for Environmental Health; CDC; Division of HIV Prevention; National Center for HIV, Viral Hepatitis, STD, and Tuberculosis Prevention; CDC; Global Immunization Division; Global Health Center; CDC; Division of Cancer Prevention and Control; National Center for Chronic Disease Prevention and Health Promotion; CDC; Division of Emergency Operations; Office of Readiness and Response; CDC; Division of Environmental Health Science and Practice; National Center for Environmental Health; CDC; Division of Infectious Disease Readiness and Innovation; National Center for Emerging and Zoonotic Infectious Diseases; CDC; Division of Healthcare Quality Promotion; National Center for Emerging and Zoonotic Infectious Diseases; CDC; Division of Environmental Health Science and Practice; National Center for Environmental Health; CDC; Division of Jurisdictional Support; National Center for State, Tribal, Local, and Territorial Public Health Infrastructure and Workforce; CDC; Office of Public Health Practice; National Center for Chronic Disease Prevention and Health Promotion; CDC.

## Abstract

Program evaluation is a critical tool for understanding and improving organizational activities and systems. This report updates the 1999 CDC Framework for Program Evaluation in Public Health (*CDC. Framework for program evaluation in public health. MMWR Recomm Rep 1999;48[No. RR-11];1–40*) by integrating major advancements in the fields of evaluation and public health, lessons learned from practical applications of the original framework, and current Federal agency policies and practices. A practical, nonprescriptive tool, the updated 2024 framework is designed to summarize and organize essential elements of program evaluation, and can be applied at any level from individual programs to broader systems by novices and experts for planning and implementing an evaluation. Although many of the key aspects from the 1999 framework remain, certain key differences exist. For example, this updated framework also includes six steps that describe the general process of evaluation planning and implementation, but some content and step names have changed (e.g., the first step has been renamed Assess context). The standards for high-quality evaluation remain central to the framework, although they have been updated to the five Federal evaluation standards. The most substantial change from the 1999 framework is the addition of three cross-cutting actions that are core tenets to incorporate within each evaluation step: engage collaboratively, advance equity, and learn from and use insights. The 2024 framework provides a guide for designing and conducting evaluation across many topics within and outside of public health that anyone involved in program evaluation efforts can use alone or in conjunction with other evaluation approaches, tools, or methods to build evidence, understand programs, and refine evidence-based decision-making to improve all program outcomes.

## Introduction

Program evaluation is a critical function that communities and organizations should undertake to improve and strengthen their activities and systems (*1*). As a scientific activity, program evaluation uses systematic data collection and analysis of programs, policies, and organizations to assess their effectiveness and efficiency (*2*–*4*). Evaluation can provide insights to many questions, including the strengths of current programs and areas for improvement throughout a program life cycle, such as the adequacy of program resources, accuracy of program assumptions, quality or fidelity of program operations, and the intended and unintended effects of a program (*5*).

Although program evaluation often uses methods that are also used in research, the purpose is different. Whereas research primarily aims to contribute to generalizable knowledge, evaluation aims to continuously improve programs and organizations and produce findings and recommendations for decision-making (*4*,*6*). Program evaluation can help clarify how to improve existing programs and build upon their strengths, why a program is or is not being implemented as planned or producing intended results, and why certain trends or patterns are observed in existing data sources.

The understanding of evaluation and its importance for improving programs has increased over the past several decades. At the Federal level, the Foundations for Evidence-based Policymaking Act of 2018 (Evidence Act) prioritizes and “elevates program evaluation as a critical agency function,” and recognizes that “pressing challenges face our nation today, with urgent needs for evidence about the approaches that work best to … address current and future challenges” (*4*). As such, Federal agencies are investing in building evaluation capacity and focusing more efforts on evaluating their programs and activities and using the findings about program processes and outcomes for decision-making and continuous program improvement. Program evaluation is most effective when it is appropriately resourced and fully integrated into the entire lifecycle of a program (i.e., program design to program conclusion) (*7*).

CDC’s program evaluation portfolio has been guided by the 1999 Framework for Program Evaluation in Public Health (*8*) (the 1999 framework). The 1999 framework is a “practical, nonprescriptive tool, designed to summarize and organize essential elements of program evaluation” (*8*). It placed evaluation within the broader context of public health practice and described a clear six-step process for conducting evaluation as well as four central standards to guide the production of high-quality evaluations (*9*). The original framework also represented a major milestone for evaluation practice at CDC by providing a central organizing process for planning and implementing evaluations that could be used by agency programs both to inform their own evaluative efforts and to provide guidance to funding recipients about the agency’s expectations for how evaluations would be performed (*6*). Since its publication, the 1999 framework has been cited in approximately 300 peer-reviewed articles and has grown from an internal tool used to guide effective program evaluation within CDC to a foundational element of projects worldwide, reaching approximately 50 countries on six continents. In addition, the application of the framework has extended beyond public health into areas such as clinical research, education, and the military (*6*,*10*,*11*).

CDC recognized the need to update the original CDC framework with advancements in the fields of evaluation and public health over the past 25 years; integrate lessons learned from practical applications of the framework; and align with current Federal agency policies, practices, and priorities (*3*,*4*,*12*–*16*). To reflect the current state of program evaluation and extend the framework’s value and use, CDC updated the framework through a multiyear process. Two key principles guided the questions, discussions, and decisions that informed this update: 1) maintain the practicality and simplicity of the framework and 2) refresh the framework rather than engage in a wholesale change. The widespread use and application of the original framework across many different contexts suggested that many aspects of the framework were useful. Thus, the updated framework retains the aspects that guided evaluations over the past several decades and includes modifications and new materials that allow evaluators continued flexibility in applying the framework across a wide array of contexts, including those that are emerging and evolving.

## Methods for Updating the Program Evaluation Framework

The process for updating the framework included gathering information and insights from various evaluation framework users, including input from Federal and non-Federal evaluators, and a literature review of evaluation and public health publications. The methods and procedures are described briefly here, and additional information is available (https://stacks.cdc.gov/view/cdc/160381)*.* To guide the process, an 80-person Evaluation Framework Work Group was convened, comprising CDC staff with expertise in evaluation representing a range of CDC programs. A subgroup of volunteers from the larger work group formed a steering committee with 21 members that guided development and implementation, reviewed results, discussed and determined revisions, and contributed to revising the framework. Feedback on framework drafts was gathered from reviewers within and outside CDC and incorporated into the final document.

Groups of framework users were identified. These groups and persons provided information to understand how the framework was adapted and used in different settings, what aspects of the framework were useful, challenges experienced when using the framework, and gaps identified in the framework’s content. A mixed methods approach for gathering information included in-depth interviews, surveys, listening sessions, and a request for public comment (*17*). Data were collected during March 2022–February 2023 from approximately 850 groups or persons who had experience using the evaluation framework. Information was collected through a survey of Federal employees and staff (n = 123) within and outside of CDC, virtual listening sessions with approximately 450 CDC-funded recipients and evaluators working with members of American Indian or Alaska Native communities (n = 172), telephone interviews with Federal evaluation leaders (n = 11) from seven Federal agencies, relevant responses to a public request for information (n = 22), a virtual session with work group members (n = 53), and attendees at a CDC Evaluation Day session (n = 38).

Data were cleaned and analyzed in Microsoft Word and Excel, and descriptive statistics for quantitative data and thematic analysis for qualitative data were generated. Information was synthesized across all activities with major themes related to conceptual design recommendations, terminology additions or revisions, and areas for additional detail or clarity.

A literature review was conducted to identify key conceptual evaluation advancements to include in the framework. The literature review included English language scholarship and practice published during 2013–2023 in a purposive sample of evaluation (n = 15) and public health journals (n = 2) selected by the steering committee that were indexed in Scopus and the Education Resources Information Center library. Search criteria included 41 search terms in article title, abstract, or keywords related to the cross-framework actions and steps. Results of the literature search yielded 3,436 publications from Scopus, 290 of which were analyzed for full-text review. Data were extracted using Covidence literature management software and analyzed in Excel to identify key themes. This activity was reviewed by CDC, deemed not research, and was conducted consistent with applicable Federal law and CDC policy.*

## Defining Key Concepts

Before describing the updated framework in detail, multiple key definitions are provided to facilitate a common understanding of the program evaluation framework content:

- **Evaluation:** An assessment using systematic data collection and analysis of one or more programs, policies, and organizations intended to assess their effectiveness and efficiency (*2*).
- **Program:** Any set of related activities undertaken to achieve an intended outcome (*18*). In this context, program is used to describe the object of evaluation, which could be any organized public health action. This definition is deliberately broad because the framework can be applied to almost any organized public health activity including interventions, surveillance systems, policy development and implementation, outbreak investigations, emergency response efforts, laboratory diagnostics, mass media and communication initiatives, infrastructure projects, training and educational activities, community efforts, research initiatives, and systems (*8*).
- **Interest holder:** Any person or organization having an investment in the evaluation, such as those served or affected by the program, those planning or implementing the program, those who might use the evaluation findings, and those who are skeptical about the program. Previously referred to as “stakeholder” (*8*), a term that can indicate a power differential between groups and that is recognized as having a violent connotation for certain American Indian or Alaska Native tribes and tribal members (*19*–*22*). Advancing equity requires many actions, one of which is using inclusive and respectful language in communications. Replacing the term stakeholders aligns with an equity-centered approach to communications because it recognizes the cultural, linguistic, environmental, and historical experiences of the many audiences of this evaluation framework and persons who might be affected by use of the framework (*19*,*23*,*24*). Stakeholder was replaced with interest holder to emphasize that anyone with an interest in the evaluation or program that is the subject of the evaluation are to be engaged in this collaborative process.
- **Performance measurement:** Ongoing monitoring and reporting of program accomplishments, particularly progress toward pre-established goals (*4*).
- **Standards:** Factors that guide evaluation decisions and are used to determine what constitutes high-quality program evaluation (*16*).
- **Health equity:** The state in which everyone has a fair and just opportunity to attain their highest level of health. Achieving this requires ongoing societal efforts to address historical and contemporary injustices; overcoming economic, social, and other obstacles to health and health care; and eliminating preventable health disparities (*14*).

## Types of Evaluation

Various evaluation terms are used and many types of evaluation can be applied in different contexts, which are continuously evolving. Summarizing all types of evaluation is beyond the scope of this report. Certain key types of evaluation are as follows:

- **Formative evaluation:** Assesses whether a program, policy, or organizational approach, or some aspect of these, is feasible, appropriate, and acceptable before it is fully implemented. It can include process and outcome measures (*4*).
- **Process or implementation evaluation:** Assesses how the program, intervention, operation, or regulation is implemented relative to its intended theory of change. It often includes information on processes, content, quantity, quality, and structure of what is being assessed (*4*).
- **Outcome evaluation:** Measures the extent to which a program, policy, or organization has achieved its intended outcome(s). It cannot attribute causality (*4*).
- **Impact evaluation:** Estimates and compares outcomes with and without the program, policy, or organization, usually seeking to determine whether a causal relation can be established between the activity and the observed outcomes (*4*).
- **Economic evaluation:** Examines program effects relative to the costs of the program. Common approaches include cost analysis, cost-benefit analysis, cost-effectiveness analysis, and cost-utility analysis. It might overlap with other evaluation types depending on the evaluation question(s) and type of economic evaluation used (*25*).

## Program Evaluation Framework

The program evaluation framework guides the design and implementation of high-quality evaluations by providing a structure that summarizes and organizes the essential elements of program evaluation. The framework can be applied at any level: a single intervention, multicomponent interventions, programs comprising multiple projects, and even broader systems. In addition, the framework can be used in conjunction with other frameworks from within or outside of public health.

**FIGURE 1:**
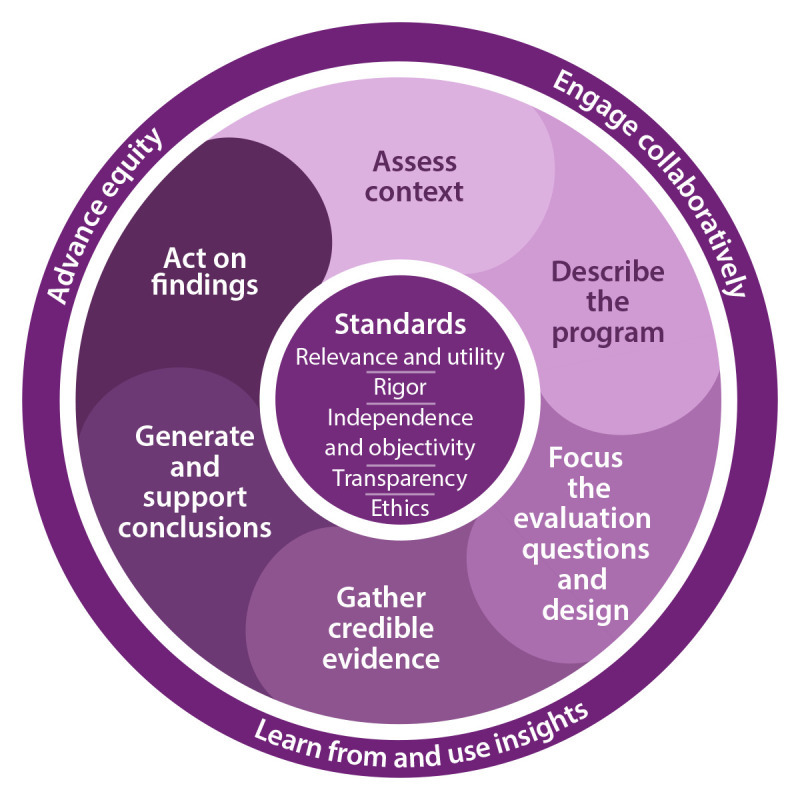
CDC Program Evaluation Framework including three cross-cutting actions, six evaluation planning and implementation steps, and five evaluation standards

Although much of the updated framework is similar to the previous version, certain key differences exist (Table 1). Like the original, the updated version includes six steps that describe the general process of evaluation planning and implementation and a set of evaluation standards that describe what constitutes high-quality evaluation. Some step names and content have changed and been updated, and the first step is new. The standards remain central to the framework, although they have been changed to the Federal evaluation standards (*16*). The most substantial change from the original framework is the addition of cross-cutting actions, which represent three core tenets to be addressed in each framework step: 1) engage collaboratively, 2) advance equity, and 3) learn from and use insights. The remainder of this report describes in detail the Federal evaluation standards, the cross-cutting actions, and each step (Figure 1) (Box 1).

**TABLE 1:** Comparison of 1999 CDC Framework for Program Evaluation in Public Health* with 2024 CDC Program Evaluation Framework

Characteristic	1999 framework	2024 framework
Evaluation steps	Engage stakeholders Describe the program Focus the evaluation design Gather credible evidence Justify conclusions Ensure use and sharing lessons learned	Assess context Describe the program Focus the evaluation questions and design Gather credible evidence Generate and support conclusions Act on findings
Cross-cutting actions	—	Engage collaboratively Advance equity Learn from and use insights
Standards	Utility Feasibility Propriety Accuracy	Relevance and utility Rigor Independence and objectivity Transparency Ethics

* **Source:** Milstein RL, Wetterhall SF; CDC Evaluation Working Group. Framework for program evaluation in public health. MMWR Recomm Rep 1999;48[No. RR-11]:1–40.

BOX 1 **2024 CDC Program Evaluation Framework cross-cutting actions, steps, and standards**

**Table:** 

**Cross-cutting actions: incorporate core tenets into each framework step** Engage collaboratively: facilitate co-ownership of the evaluation with interest holders to improve collaboration and input on decision-making. Advance equity: contribute to advancing equity and eliminating health inequities by using collaborative and equitable evaluation approaches. Learn from and use insights: learn throughout the evaluation about the program and evaluation itself and use these insights for improvement and decision-making. **Steps: describe the general process of evaluation planning and implementation** Assess context: understand the context including readiness for evaluation, interest holders, place, and evaluation capacity. Describe the program: create narrative description (need, inputs, activities, outcomes, contextual factors, and stage of development) and logic model or program roadmap (activities and short-term, intermediate, and long-term outcomes). Focus the evaluation questions and design: purpose, evaluation type, intended users and use, evaluation questions, and evaluation design. Gather credible evidence: establish expectations, methods, indicators, data sources, data quantity and quality, data collection, and context considerations. Generate and support conclusions: analyze, interpret, and make recommendations. Act on findings: plan, prepare findings for use, and facilitate insights into action. **Standards: guide what constitutes high-quality evaluation** Relevance and utility: address questions of importance and serve the information needs of interest holders. Rigor: produce findings that interest holders can confidently rely on while providing clear explanations of limitations. Independence and objectivity: strive to be as independent and objective as possible for interest holders, experts, and the public to accept the findings. Transparency: ensure transparency in the planning, implementation, and reporting phases to enable accountability and help ensure that aspects of an evaluation are not tailored to generate specific findings. Ethics: conduct evaluation to the highest ethical standards to maintain trust in the efforts.

### Program Evaluation Standards

Evaluation standards such as relevance and utility, rigor, independence and objectivity, transparency, and ethics are designed to improve the quality of evaluations by guiding decisions made in each step of the planning and implementation process. These standards are not intended to be applied as rigid rules and are intentionally broad to give evaluators and interest holders the flexibility to consider their unique circumstances, weigh the various options, and determine the best course of action. Depending on circumstances, the importance of one evaluation standard might need to be balanced with the relative importance of another standard, and decisions made based on this balance might need to be revisited if the relative importance of the standards changes during an evaluation.

Federal evaluation practice adheres to five broad evaluation standards that are part of the Evidence Act implementation and described in detail in the Office of Management and Budget M-20–12 (*16*). These are similar to evaluation standards used by other organizations (*26*). The standards are intentionally broad to provide flexibility to adapt to the unique circumstances, weigh the various options, and determine the best course of action. Flexibility should not be interpreted as ignoring or considering options that are contrary to the evaluation standards. Because evaluation is a scientific activity, those conducting evaluations should “uphold scientific integrity as they undertake evaluation activities” (*4*).

The evaluation standards summarized in this report reflect current thinking on evaluation standards and practices (*16*,*27*). They might be updated to address changes in the evaluation landscape and to incorporate new practices developed over time (*16*). These standards are complementary to, and can be used in conjunction with, evaluator competencies (*15*), evaluator guiding principles (*13*), cultural competence statements (*12*), and other evaluation standards (*26*) to ensure robust evaluation practice.

#### Relevance and Utility

Evaluations should address questions of importance and serve the information needs of interest holders to be useful. Findings should be actionable; available in time for use; and presented in ways that are understandable, culturally responsive, and informative for taking action (e.g., budgeting, program improvement, accountability, management, regulatory action, policy development, and strategic planning).

#### Rigor

Evaluations should produce findings that interest holders can confidently rely on while providing clear explanations of limitations. The rigor of an evaluation depends on thoughtful planning and implementation of the underlying design and methods as well as how findings are interpreted and reported. Credible evaluations should be planned, implemented, and interpreted by qualified evaluators in collaboration with interest holders. These evaluators should have the relevant education, skills, and experience for the methods undertaken. An evaluation should use the most appropriate design and methods to answer the evaluation questions while balancing the evaluation goals, scale, timeline, feasibility, and available resources.

#### Independence and Objectivity

Evaluations should strive to be as independent and objective as possible for interest holders, experts, and the public to accept their findings. The implementation of evaluation activities should be appropriately insulated from political and other undue influences that might affect their objectivity, impartiality, and professional judgment. Evaluators should strive for objectivity in the planning and conducting of evaluations and in the interpretation and dissemination of findings, avoiding conflicts of interest, bias, and any other partiality. To enhance objectivity, evaluators can regularly assess their potential biases, which might affect whom they choose to engage in an evaluation, to what they pay the most attention, and what they might be overlooking as a result.

#### Transparency

Evaluations should be transparent in the planning, implementation, and reporting phases to enable accountability and help ensure that aspects of an evaluation are not tailored to generate specific findings. Decisions about the evaluation’s purpose and objectives, the range of interest holders who will have access to details of the work and findings, the design and methods, and the timeline and strategy for releasing findings should be clearly documented before conducting the evaluation. Once evaluations are complete, comprehensive reporting of the findings should be released in a timely manner and provide sufficient detail so that others can review, interpret, replicate, or reproduce the work.

#### Ethics

Evaluations should be conducted to the highest ethical standards to maintain trust in the process and products. Evaluations should be planned and implemented to safeguard the dignity, rights, safety, and privacy of participants and other interest holders or affected entities. Evaluators should abide by current professional standards pertaining to treatment of participants. Evaluations should be equitable, fair, and just and should account for cultural and contextual factors that could influence the findings or their use.

### Cross-Cutting Actions

How evaluators and interest holders approach the act of evaluation is as important as what steps are used to carry out the evaluation process. The following three cross-cutting actions are core tenets of evaluation practice that need to be addressed within each of the steps: 1) engage collaboratively, 2) advance equity, and 3) learn from and use insights. Intentionally integrating each of these actions throughout an evaluation is more likely to produce rigorous evidence that is informed by multiple perspectives derived from both lived and professional experiences; generate insights that improve understanding about how to design and implement public health activities that further health equity; and provide evidence that is meaningful, informative, timely, and actionable. In addition, engaging in evaluations that embrace collaboration, welcome all voices, and create safe environments that support learning from both successes and failures can help to instill ongoing reflection and continuous improvement that are rooted in equitable processes.

The cross-cutting actions described in this section are depicted as wrapping around the framework steps (Figure 1). This is intentional because these actions affect how all six steps are performed. As such, though these actions are described, their use also is integrated into the discussion of each step later in the document.

#### Engage Collaboratively

Central to the framework is facilitating co-ownership of the program evaluation with interest holders to improve collaboration and input on decision-making. This collaboration starts at the beginning of the evaluation planning process and continues into the implementation and interpretation phases. The process actively engages persons with a broad range of perspectives derived from both lived and professional experiences. Within each step of the evaluation process, evaluators are responsible for intentionally creating an environment that is open to and respects all views by building trusting relationships and encouraging active sharing and listening to ideas among all parties involved (*15*).

Collaborative approaches to evaluation can have multiple benefits. For example, engaging a diverse set of interest holders can improve the program evaluation process and outcomes by providing a more complete understanding of the context, including its complexities (*28*), and help decrease overemphasis on values held by specific persons or groups (*29*). In addition, this approach can increase the validity of findings (*30*) and improve the likelihood that results are used by interest holders (*30*–*32*). Persons reviewing the evaluation findings might view the results as less credible if interest holders are not engaged in the evaluation (*33*) in a manner that respects and integrates their perspectives (*34*). Engaging interest holders has the additional potential benefit of improving the evaluation capacity of the persons who take part (as well as the organizations with which they are affiliated) because of learning through hands-on experiences (*30*,*35*,*36*).

The extent to which interest holders are involved directly in planning and implementing the program evaluation will vary and depend on factors such as interest holder availability (*30*,*37*). Collaboratively engaging interest holders in evaluation might require a shift in thinking among evaluators and those involved with evaluations toward the allocation of sufficient time for engagement and inclusion throughout an evaluation (*38*). Evaluations that fully engage interest holders in true collaboration often require more time (*30*), so evaluators need to consider how to best balance providing timely evidence for decision making while ensuring adequate time for collaborative engagement. In addition, collaborative evaluations require that evaluators effectively facilitate power differentials and dynamics among types of interest holders and between the evaluator and the interest holders (*30*).

Respecting and understanding the availability of collaborators throughout the evaluation process is important. Persons who can collaborate at the beginning of an evaluation might not be available throughout the entire evaluation, as contexts and circumstances might naturally change. Evaluators are responsible for communicating with collaborators throughout the evaluation to determine whether the level and type of collaboration continue to be effective for those involved while maintaining a range of perspectives. Engaging in evaluation planning and implementation processes requires commitment, and it is important that a person’s time and expertise are valued appropriately within the context.

#### Advance Equity

Evaluation can contribute to advancing equity and eliminating health inequities in multiple ways (*14*). First, by using collaborative and equitable evaluation approaches, evaluators can create environments where everyone is respected and heard (*39*). Such environments can advance equity by creating forums where interest holders who might otherwise not have been as involved are able to share their perspectives (*14*,*29*,*30*). Evaluators should be attentive to the range of interest holder perspectives and the diverse (and sometimes divergent) input that might arise, understand how power differentials between interest holders can influence equity in the evaluation process (*28*), and consider how to balance these perspectives and power differentials. Including members of typically underrepresented groups (e.g., affected communities) and ensuring that they be represented, involved, and heard within the evaluation development, implementation, and interpretation phases is important, with lived experiences welcomed and valued alongside technical or professional experiences. When engaging with different interest holders, employing equitable communication principles throughout the evaluation can reduce bias in language and enable positive and constructive interactions (*40*).

Second, in each step of the framework and when applying the standards, evaluators and interest holders can think about how to advance equity and potential effects of decisions. Doing so can create an intentional process in which evaluation discussions can provide insights about how the program can address drivers, which are “factors that create, perpetuate, or exacerbate a health inequity” (*14*,*39*). For example, when describing the program (Step 2), evaluators can ask questions about how the program’s underlying theory of change addresses the drivers of health inequities. If the theory of change does not address these topics, interest holders can discuss what opportunities exist. In addition, care should be taken during evaluation planning to ask key evaluation questions, data collection methodologies, and data analyses (Steps 3–5) that explore and examine drivers of health inequities (*14*,*41*). These actions can produce evaluation findings that provide valuable insights about how the public health program is already contributing to health equity or how it can be adjusted to facilitate change.

Third, evaluators can conduct evaluation in a culturally responsive way that recognizes “the shared experiences of people, including their languages, values, customs, beliefs, and mores ... worldviews, ways of knowing, and ways of communicating” (*12*,*28*). Culturally responsive evaluation integrates the uniqueness of each context into the design and implementation of an evaluation, including the history, systems, and structures that can contribute to health inequities. In understanding and respecting different cultures within evaluation, it is important to acknowledge that the data provided for an evaluation depict the experiences of communities. As such, evaluators can consult with communities on the best approaches to share their story with others (e.g., release of findings), incorporate ways to benefit those providing insights throughout the evaluation and reciprocate their contributions throughout the evaluation, collaboratively interpret evaluation findings, and review communications products with interest holders so they appropriately reflect the context and avoid unintended bias (*19*). In addition, it is important to be aware of, and adhere to, the specific rights, rules, and procedures that exist within certain contexts relating to data collection; data ownership; and how data, knowledge, and insights are shared (e.g., tribal data sovereignty) (*42*).

Finally, evaluators should continuously consider how their professional and lived experiences might affect what they see and hear, their decisions, and how they engage with others in each context (*12*,*43*–*45*). Doing so can help evaluators “better understand how their own backgrounds and other life experiences serve as assets or limitations in the conduct of an evaluation” (*12*) and bring awareness to who or what they might inadvertently pay the most attention to in various contexts, how they might hear or interpret information shared by others, and generally engage more effectively with interest holders (*46*).

#### Learn From and Use Insights

Evaluations are conducted to provide results that inform decision making (*2*,*16*,*36*). Although the focus is often on the final evaluation findings and recommendations to inform action (Step 6), opportunities exist throughout the evaluation to learn about the program and evaluation itself and to use these insights for improvement and decision making (*47*). Evaluators have an important role in facilitating continuous learning, use of insights, and improvement throughout the evaluation (*48*,*49*). By approaching each evaluation with this role in mind, evaluators can enable learning and use from the beginning of evaluation planning. Successful evaluators build relationships, cultivate trust, and model the way for interest holders to see value and utility in evaluation insights. This takes dedication, skill, and perseverance on the part of the evaluator. The many aspects of facilitating learning and use with interest holders include planning for use of findings; sharing, discussing, and interpreting insights during the evaluation process (rather than waiting until the end); and discussing how to implement recommendations. These types of activities can be integrated into the evaluation plan at the beginning of an evaluation process to ensure these opportunities for learning occur (*47*).

Interest holders can experience changes in thinking and behavior from engaging in the process of planning and implementing an evaluation, including learning from and using insights throughout the process (*36*). For example, when newcomers to evaluation begin to think evaluatively, fundamental shifts in perspective can occur (*50*). Evaluation prompts staff to clarify their understanding of program goals, and this clarity might allow staff to function more cohesively as a team with a shared vision of the common endpoint(s). Immersion in the logic, reasoning, and values of evaluation can lead to lasting effects (e.g., basing decisions on data and insights instead of assumptions). As interest holders become more familiar with the evaluation process, they might develop new knowledge about evaluation, acquire additional evaluation skills, and place increased importance on the value of evaluation (*30*,*36*).

Changes in attitudes, knowledge, and skill among persons also might translate into changes within the organization, such as developing an infrastructure (e.g., systems, structures, policies, and procedures) that is more supportive of evaluation or enhancing an organization’s evaluation culture as more persons engage in evaluative thinking by regularly questioning program assumptions and asking questions such as what is working, for whom, and under what conditions (*36*,*51*,*52*). Such changes at the individual and organizational levels represent enhancements in evaluation capacity and can be an expressed intention of engaging in a collaborative evaluation process (*30*,*35*,*51*,*53*,*54*). The benefits that arise from these and other uses provide further rationale for initiating evaluation activities at the beginning of a program and ensuring evaluators and interest holders learn from and use insights throughout the evaluation.

Evaluators and interest holders also can take the following actions to increase the likelihood that evaluation findings will be useful and used:

- Ensure that interest holders are engaged in the evaluation and their perspectives and needs are understood.
- Consider evaluation use from the beginning of the process.
- Clarify who is likely to use the findings and for what purposes.
- Conduct the evaluation flexibly (i.e., modify methods and approach to align with changing or emerging needs).
- Share findings in time to inform decision making and in a manner that is responsive to the decision-making context (*32*,*36*).

### Program Evaluation Steps

The framework is composed of six steps that are important to use in any evaluation to improve how evaluations are conceived and conducted. Each step is considered when planning an evaluation and revisited during evaluation implementation. Although the steps are arranged in a linear sequence, all steps are highly interdependent, and might be encountered in a nonlinear sequence. Program evaluation, like these steps suggest, is an iterative process. For example, while formulating evaluation questions (Step 3), evaluators can consider how audiences might act on findings (Step 6) so the answers provided through the evaluation will be more useful. Earlier steps provide the foundation for subsequent progress; however, it is often the case that contextual constraints or nuances are revealed only in later steps (e.g., Steps 4 and 5) and might require revisiting and revising decisions made in earlier steps (e.g., Step 3). Furthermore, the evaluation standards and cross-cutting actions are important to consider in each step; examples of ways to apply both are provided at the end of each step.

#### Step 1: Assess Context

A first step in the evaluation process is understanding the context in which the program operates (*43*,*55*). Contextual details can include various features or components of settings in which evaluation occurs, such as location and environment; persons and their values or beliefs; political, economic, cultural, and historical circumstances; how power and privilege manifest in context; and other underlying factors that contribute to the condition(s) the program was designed to address (*12*,*15*,*56*).

Understanding the context sets the stage for a meaningful, actionable, and culturally responsive evaluation as it provides essential insights for understanding what is most important to know about the program, what constitutes program success, when evaluative insights are needed, and who needs to learn from the insights. In addition, a deeper understanding of the context can increase the validity of evaluations (*57*,*58*) and provide essential insights for interpreting the evaluation findings correctly and formulating a feasible action plan. This section describes four factors to consider when assessing context: 1) readiness for evaluation, 2) interest holders, 3) place, and 4) evaluation capacity.

**Readiness for evaluation.** Before starting an evaluation, consider whether the appropriate conditions exist to conduct an evaluation that is likely to produce relevant, useful, and rigorous insights (*59*,*60*). Evaluability assessments are a tool for examining evaluation readiness and might be particularly helpful for public health programs (*61*). Various factors can be examined through an evaluability assessment, including the extent to which the goals of the program are clearly articulated and potentially attainable through the proposed activities, the clarity of the program theory (e.g., how the proposed activities are envisioned to lead to the desired programmatic results), the availability of appropriate resources to support the proposed evaluation (e.g., funding, staff member availability, and data), and the existing level of interest in the proposed evaluation (*43*). Multiple factors examined in an evaluability assessment could be reviewed at the start of a program’s lifecycle and addressed to ensure or increase the likelihood of readiness when a program or organization needs to begin an evaluation.

Evaluability assessment findings might indicate that it would be helpful for the program to reach a common understanding of how the program is envisioned to work before engaging in an evaluation. Ensuring consensus on the underlying theory and how the program will proceed from activities to outcomes might create better conditions for describing the program (Step 2), developing clearer evaluation questions (Step 3), and defining what to measure through data collection efforts (Step 4). The assessment might suggest that although persons who are most likely to make use of the evaluation are specifically interested in learning whether the program has produced the intended outcomes, that it is too early in the program’s lifecycle for the intended outcomes to occur. Such a finding would suggest that it is appropriate to refocus the purpose of the evaluation on program implementation rather than outcomes (*43*). Alternatively, the assessment could show that conditions are optimal and the evaluation can proceed.

**Interest holders.** Ensuring an evaluation is relevant and useful requires understanding who is invested in, and might be affected by, the evaluation (*16*). To increase the likelihood that the perspectives of persons with a broad range of professional and lived experiences are included in the evaluation, it can be helpful to assign persons or groups to the following categories. Persons might be assigned to more than one category.

- **Persons served or affected by the program.** These interest holders include persons or organizations affected by the program, either directly (e.g., receive services) or indirectly (e.g., benefit from enhanced community assets). They have a vested interest in the evaluation because changes made to the program that are based on the evaluation findings might alter how they experience the program and what benefits they derive.
- **Persons who plan or implement the program.** These persons have a vested interest in the evaluation because they might need to modify the program based on the evaluation findings. Although the efforts of various groups and persons (e.g., program funders, staff members who engage in day-to-day program operations, and partners who support programming) contribute collectively to program delivery, they are not necessarily a single interest group. Subgroups of persons and organizations involved in program delivery can have different perspectives and might have alternative agendas.
- **Persons who might use the evaluation findings.** These persons learn from, and act on, the evaluation findings. This is a broad category that includes persons or groups who have the authority to make decisions regarding the program (e.g., can adjust funding allocations or modify program processes) or have a general interest in the results because they design, implement, evaluate, or advocate on behalf of the program being evaluated or similar programs. The primary intended users of the evaluation, those who have a specific interest in the evaluation and clear idea for how to use the findings, should also be considered and highlighted (*36*). Consider identifying primary intended users early in the development of the evaluation and maintain frequent interactions with them so the evaluation addresses their unique information needs (*36*) while also integrating the perspectives and values of other categories of persons identified in this section.
- **Persons who are skeptical about the program.** These persons or groups are skeptical or antagonistic toward the program. Opposition to a program might stem from differing values regarding what change is needed or how to achieve it. When these types of opposition exist, it might be helpful to engage the help of program opponents in the inquiry to strengthen the evaluation’s credibility. These contrasting views also might reveal additional needs and uses for the evaluation.

When assessing the context, it is important to understand the different perspectives or values that the identified persons hold about the program and what might be examined through the evaluation. Persons might view program activities and outcomes differently (Step 2), propose different questions to answer in the evaluation resulting from their unique perspectives (Step 3), value different types of designs and methods (Steps 3 and 4), and differ in their views regarding what constitutes programmatic success and how to interpret (Step 5) and act upon the evaluation findings (Step 6).

**Place.** Well-designed evaluations recognize, acknowledge, and integrate the uniqueness of the place-based context in which the program and evaluation are conducted. The place dimensions can include program and community history, power dynamics, and the systems and structures that exist, and how these factors intersect with the current-day realities of marginalized communities (*28*). Aspects of the context that are important to understand, include but are not limited to (*43*) the following:

- **Program features.** These features can include why the program was developed, where it operates, how it came into existence, the specific needs the program intends to address, who it intends to serve, how it operates (including the funding mechanisms used), who is directly involved in delivering the program, who has the authority for decision-making, and who and what might influence their decision-making process (*36*).
- **Program environment.** Environmental aspects include the current and historical features of the environment in which the program operates (physical and virtual). Specific topics of importance might include the “historical, economic, health, and social dimensions of the communities” (*43*) and can highlight the strengths and assets (including the talent and expertise) of persons who interact with the program (*46*). As part of this assessment, it is important to understand how power is distributed among persons who interact with or influence the program, or who might be engaged in the evaluation (e.g., evaluation funders, planners, implementers, and users of findings). For example, understanding whose perspectives have and have not been previously heard or included provides valuable insights for considering how to engage persons in the evaluation. To advance health equity through evaluation, it is also important to understand what health inequities exist within the program environment as well as the drivers of these inequities (*14*). Such an understanding might inform the role that the program plays in the pathway between drivers and health inequities (Step 2), what elements an evaluation examines (Step 3) and how (Steps 3 and 4), and how the findings from an evaluation are interpreted and acted on (Steps 5 and 6).

**Evaluation capacity.** Understanding the program’s existing capacity to “do and use” evaluation can be helpful in engaging with persons in a way that takes into account their current understanding and valuing of evaluation (*53*). In addition, assessing existing capacity can help in identifying the strengths persons and organizations involved in the evaluation might bring to support the planning and implementation of the evaluation, as well as sharing, learning from, and using the findings (*36*,*53*,*62*). Evaluation capacity can be examined at the organizational and individual levels (*54*,*63*).

- **Organizational.** The organization(s) involved in the program (and therefore potentially the evaluation) have an important role in facilitating a high-quality evaluation. The process can benefit from evaluators understanding early in the process an organization’s capacity to support the evaluation so they can leverage existing strengths and, if needed, identifying gaps that might need to be filled to support evaluation activities. Example questions evaluators might ask about existing organizational evaluation capacity to support the conduct and use of evaluations include the following (*54*,*64*) (Table 2):

**TABLE 2:** CDC Program Evaluation Framework (Step 1 — Assess context): example questions to consider when applying cross-cutting actions and evaluation standards

Example questions	Cross-cutting action		
	Engage collaboratively	Advance equity	Learn from and use insights
In what ways are the evaluation team positioned or not positioned to engage in evaluation within this context given their own experiences (professional and lived)?	Relevant	Relevant	Relevant
Who is invited to engage in the evaluation? Are persons with varied perspectives and experiences invited to collaborate? What strengths and contributions will different interest holders bring to the evaluation? Are they available and interested in contributing in this manner? How will they be recognized or appreciated for their engagement?	Relevant	Relevant	—
What are the norms of the community or communities where the program is conducted? How do persons interact with each other and communicate? What conduct is viewed as respectful and how is trust established? What norms exist in this context regarding information sharing and learning? What languages, formats, and communication styles work best?	Relevant	Relevant	Relevant
What are the strengths of the persons and communities in which the program operates and for whom it is intended to benefit? What disparities and power differentials exist (historically and currently)?	—	Relevant	—
How to apply evaluation standard	Evaluation standard		
Consider the context at the beginning to provide a foundation for designing an evaluation that answers questions that are relevant and meaningful, delivers trustworthy findings, and provides insights on time in an actionable format. Understanding the context facilitates awareness of how evaluation findings can be used, opportunities for sharing and learning from evaluation insights, and challenges in moving from findings to action so these can be acknowledged early on and addressed during the evaluation process.	Relevance and utility		
Assess the context to provide a fuller picture of the environments and circumstances within which the program exists, which can affect rigor in various ways. Understanding the context provides insights about what can reasonably be expected in terms of data sources and access (e.g., appropriate timing for data collection activities which can lead to stronger data collection strategies). Furthermore, understanding the context can lead to selecting evaluation designs and data collection methods that are culturally responsive, increasing the likelihood of participation and more accurate and reliable data.	Rigor		
Understand a context as fully as possible to provide a clear, complete picture that balances multiple perspectives and improves objectivity. Evaluators can assess their objectivity by engaging in reflective practice, regularly assessing their potential biases which can affect who they engage, to what they pay the most attention, and what they might be overlooking as a result.	Independence and objectivity		
Document clearly which interest holders were engaged in the evaluation improves transparency by showing who was involved, and who was missing. Furthermore, contextual challenges can arise during implementation resulting in a need to change the original plan. Documenting these challenges and how they affected the evaluation can facilitate a stronger understanding among interest holders regarding the trustworthiness of the evaluation.	Transparency		
Incorporate ethical guidelines as noted in Office of Management and Budget 20–12: “To ensure fair, just, and equitable treatment of all persons and affected entities, evaluators should gain an understanding of the range of perspectives and interests that individuals and groups bring to the evaluation, including those not usually represented. This includes accounting for cultural and contextual factors (e.g., languages spoken, political and social climate, power and privilege, or economic conditions) that could influence the evaluation's findings or their use.”* This understanding begins within Step 1 (Assess context) of the evaluation process.	Ethics		

* **Source:** Office of Management and Budget. Phase 4 implementation of the Foundations for Evidence-Based Policymaking Act of 2018: Program Evaluation Standards and Practice. Washington, DC: Executive Office of the President, Office of Management and Budget; 2020:15.
  - What is the organizational culture with respect to evaluation and using evaluation findings? Is there support for evaluation generally? To what extent?
  - What resources (e.g., funds, staff members, volunteers, time, technology, and data) are available to support planning and implementation of the evaluation? Are there partnering organizations (e.g., community-based organizations and health departments) that are available, willing, and able to support the evaluation?
  - Are there internal evaluation champions who can support and effectively encourage engagement in evaluation as well as communicate about the evaluation process and findings?
  - What mechanisms already exist to share products from the evaluation with others in the organization who could benefit from the evaluation (e.g., learning forums, online repositories accessible by different organizational units, and workplace collaboration tools)?
  - Are there opportunities within the organization to reflect insights that arise throughout the course of the evaluation (e.g., monthly staff meetings)?
- **Individual.** Interest holders who help plan and implement the evaluation as well as use the findings, might hold assumptions about what evaluation is (or is not) and beliefs about the usefulness of evaluation, which might come from previous experiences with evaluation (*36*). The process might benefit from inquiring about these past experiences and learning how these have shaped interest holders’ attitudes about evaluation and the extent to which they value the information evaluations can provide.

Understanding these assumptions and beliefs can help evaluators engage in more meaningful conversations about what to anticipate from the evaluation process. Discussing how interest holders understand evaluation can lead to clarity about what questions program evaluation can address and how it is similar to, and different from, other evidence-building functions (e.g., research, performance measurement, and policy analysis). For example, interest holders might have extensive experience with performance measurement or monitoring and incorrectly interpret this activity as synonymous with program evaluation. Clarifying and addressing early in the evaluation planning process how performance measurement and evaluation are related (e.g., the former potentially serving as a data source for the latter) and how the evaluation process might differ (e.g., ability to address questions about program effects with rigor) can reduce misunderstandings and areas of confusion (*65*).

Certain persons who are invested in the evaluation might not have extensive evaluation experience; however, they might still bring valuable knowledge and skills to support the evaluation activities. Evaluators should consider the interest holders’ existing evaluation capacity (e.g., knowledge and skills) when bringing them into the evaluation steps and consider opportunity areas throughout the evaluation process for further enhancing individual evaluation capacities.

**Implementation considerations.** When assessing context, and throughout the other evaluation steps, it is important that evaluators recognize and are responsive to different perspectives, cultures, and approaches (*46*). Culture is multidimensional and includes “the shared experiences of people, including their languages, values, customs, beliefs, and mores. It also includes worldviews, ways of knowing, and ways of communicating” (*12*,*28*). Implementing evaluations that appreciate and account for culture advances equity by answering evaluation questions that matter, by analyzing data collected through ways that are meaningful and understood by those responding, and by interpreting the data within the specific context (*28*). Evaluators bring their own cultural norms to the evaluation and should engage in self-reflective practices to understand how their own culture might affect what they ask, how they ask it, what they perceive, and whether they might be inadvertently favoring certain voices over others in this evaluation context (*12*,*28*,*46*,*66*).

Evaluators and interest holders need to consider that the context can change during an evaluation and be prepared to adjust accordingly (*36*). For example, at the start of an evaluation, the interest holders who are engaged might be very supportive of the evaluation and see value and utility in the potential findings. Later in the evaluation process, interest holders might change, and new interest holders might not be as supportive (or not supportive) of the evaluation. Evaluators should stay appraised of the context so they can readily identify such changes and consider in advance how their approach to collaboration includes engaging new interest holders in the process.

#### Step 2: Describe the Program

Program descriptions identify the outcomes the program intends to achieve and the key activities that are expected to lead to those outcomes. The program description is the foundation for all subsequent steps in the evaluation, and without this description, it would be challenging to design and implement an effective evaluation. The aim is to produce a program description that is clear and concise with enough detail to facilitate an understanding of the program roadmap. The roadmap often takes the form of a one-page graphic (e.g., logic model, theory of change, or rich picture) that is accompanied by a narrative explanation providing more detailed information.

Collaborating with interest holders can aid in developing a description that is comprehensive and inclusive of different perspectives while bringing clarity and program benefits beyond evaluation planning and implementation. Collaboration also can provide an opportunity for reaching agreement about what the program is doing and aims to achieve, and how the program intends to advance health equity. Evaluations conducted without agreement on key activities and outcomes might be of limited use.

**Narrative description.** Aspects to consider in a narrative description include:

**Need.** A statement of need describes the issue, challenge, or opportunity the program is intending to solve or is contributing to solving. Potential components include the nature and magnitude of the problem or opportunity, which persons or groups are affected and how they are affected (e.g., health disparities between groups), and how the need is changing and in what manner(s) (e.g., disease trends). The statement of need also can reflect existing evidence that informs the understanding of the need, any previous efforts the program has taken to address the issue, and any potentially related factors (e.g., drivers of health inequities).
**Inputs.** Inputs are the resources needed for conducting program activities, such as personnel, partners, materials, funding, equipment, data (e.g., surveillance), and the existing evidence base. Descriptions of program inputs need to convey the amount and intensity of program services and highlight areas in which there is a potential mismatch between the activities and resources available to implement those activities. Assumptions regarding base needs for the program (e.g., culturally responsive training curriculum) can be specified as inputs if they are expected to be in place or available at the beginning of the program.
**Activities.** The activities identify what the program is implementing to effect change and achieve the intended outcomes. The description can include higher level strategies as well as the activities associated with each. For example, a public health emergency readiness and response strategy might include activities such as developing and implementing readiness and response plans, developing rapid forecasting capabilities, conducting emerging disease surveillance, and coordinating outbreak and emergency response activities.
Well-designed program descriptions clearly identify whether the activities occur sequentially or simultaneously and how activities relate to each other. The description of activities clarifies the program’s hypothesized mechanism or theory of change that explains how the activities are presumed to lead to the intended outcomes (*67*). Any existing evidence supporting the mechanisms of change can be identified and cited, and evidence gaps are also identified. In addition, it is helpful to describe the intentions regarding what needs to happen with respect to program implementation to achieve the intended outcomes (e.g., essential training and skills of program implementers, program dose and duration, key characteristics of settings where the program is implemented, and characteristics of program participants) (*68*). In public health, various groups often implement activities with the intention of contributing to shared outcomes, so program descriptions also can clarify which activities are the direct responsibility of the program or related programs or partners.
**Outcomes.** The outcomes identify who or what is expected to change as a result of the program’s efforts (i.e., program effects). For most programs, outcomes can be temporally sequenced, with shorter term (often more specific) outcomes leading to intermediate then longer-term (broader) outcomes. Many potential sources exist for identifying intended program outcomes, such as a program’s mission and objectives (often shorter or intermediate outcomes), vision and goals (often longer-term outcomes), input from interest holders (e.g., program designers, participants, partners, and funders), social science or other theories (e.g., theory of planned behavior and diffusion of innovation), research, and findings from evaluations of similar programs (*69*). It is also useful to anticipate and include unintended program consequences or outcomes to the extent that is possible.
**Contextual factors.** Public health programs often operate in settings where factors outside the program exist that might affect achieving the desired outcomes. Understanding and accounting for contextual factors that might affect the program’s success is required to design a context-sensitive evaluation to account for and interpret findings accurately. Documenting these observations might be helpful for potential users of the evaluation who wish to transfer learnings from the evaluation to another context. Much of this information will have been identified in Step 1 (Assess context) but can be documented here as part of the program description.
**Stage of development.** Programs mature and change; therefore, it is important to consider a program’s stage of development in the evaluation. For example, programs that are in planning stages will differ from those that have operated continuously for a decade. The evaluation purpose and questions posed about a program (Step 3) need to align with the program’s stage of development.
In the planning stage when program activities are untested, and during the implementation stage when program activities are being field-tested and modified, a process evaluation can provide valuable insights about “how the program or service is delivered relative to its intended theory of change, and often includes information on content, quantity, quality, and structure of services provided” (*4*). Programs in a more mature stage are often well-positioned to examine the extent to which the intended program outcomes have occurred through an outcome evaluation. Programs that assess the causal impact of their activities on outcomes relative to those of a counterfactual (i.e., a condition in which the program does not take place) will conduct an impact evaluation (*16*).

**Logic model or program roadmap.** A logic model is a graphic depiction of the relation between a program’s activities and its intended outcomes or effects. It shows the sequence of events for bringing about intended change by synthesizing the main program elements into a roadmap of how the program is supposed to work (rather than how it does work, which could be a question answered through an evaluation). It reflects the underlying theory of change, showing the “if-then” relation between earlier and later activities and the connection between activities and outcomes and earlier to later outcomes (*70*).

**FIGURE 2:**
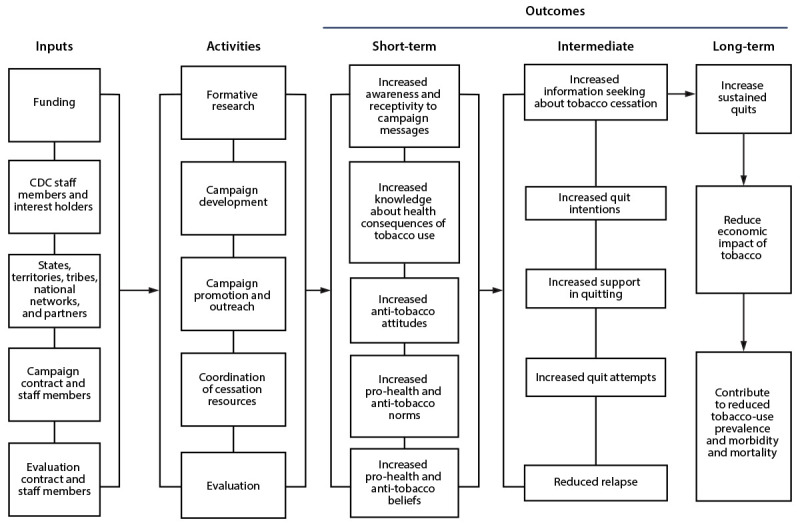
Example logic model* for Step 2 (Describe the program) of CDC Program Evaluation Framework^†^^,^^§^ * Based on CDC’s Tips From Former Smokers Campaign. R Murphy-Hoefer, CDC, personal communication, August 2024. ^†^ Contextual factors: social determinants of health, U.S. national media infrastructure, cessation support infrastructure, and other media campaigns. ^§^ Theoretical foundations: theory of reasoned action/planned behavior, health belief model, social learning theory, transtheoretical model of change (stages of change), or diffusion of innovation theory.

A simple, high-level, one-page logic model can concisely synthesize most of the program elements described above. Logic models can take many forms, though the most common elements include activities and short-term, intermediate, and long-term outcomes (and sometimes inputs) with arrows showing the connections between or among each of these (depicting the underlying theory of change). For example, a logic model was adapted from the CDC Tips from Former Smokers campaign evaluation (Figure 2).

When designed in a collaborative manner, the logic modeling process can improve the level of clarity and agreement among partners about the main strategies and activities and intended program outcomes. During these discussions, persons can work together to clarify the program’s chain of events and identify key assumptions or gaps in the logic of the program’s effects. Ensuring a clear program logic exists without apparent gaps is important to ensuring the program is ready for evaluation (*60*).

Families of logic models or nested logic models can be created to display a program at different levels of detail, from different perspectives, or for different audiences. For example, a high-level logic model could show an entire organization. A second logic model could focus on a specific program or component within the broader logic model, showing the specific activities for that program and aligning these activities with relevant programmatic outcomes. Finally, a third nested logic model could show a specific intervention within the program. Viewed together, the group of logic models can comprehensively show all aspects of a program, which can be useful for program planning and the next steps in the evaluation process.

Although logic models are a useful tool, they are not the only method to visually depict a program (*43*). The linearity of a logic model might not resonate in all contexts, so it is important that interest holders are engaged early to determine a method that will resonate within their context.

**Implementation considerations**. Logic models and the narrative that accompanies them are “living documents” and it is important to update them as program changes occur or are anticipated. A few examples of scenarios which might require logic model revisions include changes in context (e.g., pandemic or policy changes that affect the program) or inputs (e.g., unanticipated resource needs during a pandemic), advancements in knowledge or practice from research (e.g., new research suggesting proposed connections between activities and outcomes would not operate as depicted), or evaluations of the program (e.g., modifications to program activities are made in response to an evaluation’s findings).

Sometimes inconsistencies arise among collaborators in how program activities or outcomes are described and the envisioned connections among them. In addition, apparent gaps or inconsistencies in the program logic might arise such as when a particular dose or level of an activity described is unlikely to effect change in an outcome or an outcome in the presumed causal pathway is skipped (e.g., knowledge or skill development is not depicted before a behavior change that would rely on this). Often these inconsistencies can be overcome through further discussion among the interest holders or through further examination of existing evidence. Occasionally, these issues point to an underlying lack of clarity in the program logic suggesting that the program might need more design work before being ready for an evaluation (*60*,*61*); in this case, the issues need to be resolved before moving forward with the evaluation.

Collaboratively engaging with interest holders on understanding and describing the program can take various forms and could include a series of meetings to discuss and reach consensus on program descriptions, methods for receiving feedback, and working to facilitate a clear understanding and consensus. To advance equity through evaluation, these discussions also can include conversations about whether (or how) the activities advance equity, whether the program has (or could have) any long-term intended health equity outcomes, and the pathway to achieve those outcomes.

#### Step 3: Focus the Evaluation Questions and Design

After the program has been described, the next step is to determine on which parts of the program to focus the evaluation efforts. Because most parts of a program logic model or roadmap can be evaluated, this step prioritizes the information needs for learning and use. The main products of this step are the following:

- Purpose statement explaining why the evaluation is being performed, how the findings are likely to be used, and who is likely to learn from or use the findings,
- Statement about the type of evaluation that will be conducted (e.g., process and outcome),
- List of intended users and use of the evaluation findings,
- List of evaluation questions (Table 3), and

**TABLE 3:** CDC Program Evaluation Framework (Step 2 — Describe the program): example questions to consider when applying cross-cutting actions and evaluation standards

Example questions	Cross-cutting action		
	Engage collaboratively	Advance equity	Learn from and use insights
Does the group of individuals involved in helping to describe the program hold multiple, diverse perspectives (e.g., program designers, implementers, participants in the program)?	Relevant	Relevant	—
To what extent is time allocated to engage interest holders to discuss, understand, and revise the program logic and expectations? Are there opportunities to discuss the underlying logic, compare with existing evidence (e.g., evaluations of similar programs, relevant theories and research), and potentially refine the program roadmap?	Relevant	—	Relevant
In what ways is the program’s commitment to advancing equity reflected in the logic model and associated narrative? Are there opportunities for reflecting this commitment and the pathway to change more clearly and directly (e.g., through creation of a “zoom in” logic model)?	—	Relevant	—
How to apply evaluation standard	Evaluation standard		
Describe the program to provide essential information about how the program intends to operate and how this will lead to the desired changes. When a program description is clear, this can enhance the relevance of the evaluation by informing the development of evaluation questions that are appropriate to the program’s stage of development and aligned with the program’s intent.	Relevance and utility		
Develop logic models, program roadmaps, and related visual depictions to provide insights about anticipated program outcomes and what is likely to precede each outcome. These are invaluable insights when focusing the evaluation questions (Step 3) and when designing instruments to collect new data or identifying existing data to use for the evaluation (Step 4). Narrative program descriptions identify constructs that might be helpful to measure specific evaluation questions, the potential dosage of a program that might be needed to realize the desired changes, and the mechanisms of change that might be examined in an evaluation.* Such clarity can increase the likelihood that the measurements are collecting information about what the evaluation intends to measure and doing so at the appropriate time.^†^	Rigor		
Share program descriptions readily with others to explain in an accessible way what the program does and how it intends to effect change, providing clarity and transparency with respect to program intent. Components of logic models, or other visual depictions, can highlight the specific aspects of the model that the current evaluation is examining, thereby making the focus of the evaluation more transparent.	Transparency		
Develop program descriptions collaboratively to provide opportunities for interest holders to discuss program activities and intended outcomes, helping create an objective overview of the program. These discussions might highlight areas of the program to examine through the evaluation (Step 3) that create programs that are more fair, just, and equitable.	Independence and objectivity; ethics		

* **Source:** McDavid JC. Huse I, Hawthorn LR. Program evaluation and performance measurement: an introduction to practice. 3rd ed. Thousand Oaks, CA: Sage; 2018.

^†^ **Source:** Chen HT. Practical program evaluation: theory-driven evaluation and the integrated evaluation perspective. Thousand Oaks, CA: Sage; 2014.
- Description of the overarching evaluation design that will be used to answer the evaluation questions.

The objective in this step is to develop collaboratively an optimal, culturally responsive evaluation design that accommodates the program context and available resources, anticipates intended uses, and incorporates all relevant evaluation standards. A well-developed and articulated purpose statement and a clear set of evaluation questions can be referred to throughout the evaluation to help decision-making regarding how the evaluation will be conducted, analyzed, and interpreted.

Flexibility is important when focusing the evaluation because what is learned in Steps 4 and 5 might affect the decisions made in Step 3. For example, perhaps interest holders would like to answer a specific evaluation question, but in Step 4 it is discovered that the data interest holders would find most credible in answering this question cannot feasibly be collected given available resources.

**Purpose.** Articulating an evaluation’s purpose (i.e., intent and aims) can help to prevent premature decision-making regarding how the evaluation will be conducted and maintain the intended scope of the evaluation efforts. Characteristics of the program, particularly its stage of development and context, will influence the evaluation’s purpose. There are many potential purposes for conducting an evaluation, although they are all aimed at learning about and understanding a program and using the findings for program improvement. Evaluations might have more than one purpose, although it is important to gain clarity with interest holders about the highest priority purpose(s) so the scope of the evaluation does not become too broad.

The evaluation’s purpose helps to focus the evaluation by identifying the most appropriate uses of the evaluation’s findings, the types of evaluation questions likely to be within the evaluation’s scope, and the strength of evidence needed from the evaluation. Some example evaluation purposes include identifying opportunities for improving a program (*36*,*71*), providing insights in support of innovation (*36*,*72*), examining the program’s effectiveness (*36*), accountability (i.e., program oversight) (*16*,*36*), advancing human rights and social justice (*71*), and building evaluation capacity (*30*,*35*,*36*) (Table 4).

**TABLE 4:** CDC Program Evaluation Framework (Step 3 — Focus the evaluation questions and design): example evaluation purposes and potential uses for findings

Evaluation purpose	Use for finding
**Identify opportunities for improvement** Information gathered can be used to better describe program processes, improve how the program operates, fine-tune the overall program strategy, and identify what is working well to continue and expand those aspects.*^,†^	Adjust procedures to improve efficiency, and fidelity to implementation processes, program reach, or both Make modifications to improve cultural responsiveness of program processes Improve the clarity and accessibility of program products (e.g., educational materials) Adjust procedures so they are more responsive to changing contexts
**Support innovation and development** Provides ongoing insights to support innovation and continuous development of programs that do not anticipate reaching a set implementation model but rather respond and adapt to changing contexts.*	Learn about changing environment and potential responses Adapt and change implementation approach regularly Enhance evaluative thinking among partners Influence strategies and approaches to other programs
**Examine program effectiveness** Examines the relationship between program activities and observed consequences. This is most appropriate for mature programs that have a clear and stable implementation in place,* and might provide valuable insights about underlying mechanisms of change that could be leveraged in related programs.	Expand (or “scale”) program Adjust funding allocations Continue program as is Include as evidence-based or promising practice for use in future public health programming Inform training and professional development Mitigate risk to the program
**Accountability** Provides insights, typically to management, for conducting program oversight. Considerations often include how funds are allocated and spent, return on investment, number of individuals reached, whether implementation efforts are going as planned, and whether goals are achieved or on target.*^,§^	Fulfill commitments to interest holders Delve deeper into why a program is not being implemented as intended or performing as expected Request additional funds or reallocate funds to support implementation for improved performance Modify programming to increase efficiencies or alignment with original program intent
**Advance human rights and social justice** Provide insights regarding how programs are, or are not, supporting change aligned with advancing human rights and social justice.^†^	Adjust program systems, policies, and procedures to remove barriers to access Adopt program strategies that advance equitable process and outcomes Adapt program to increase the likelihood of improving equity in health outcomes
**Build evaluation capacity** Participating in evaluation planning and implementing can build evaluation knowledge and skills among interest holders. Evaluations can have a specific intent of enhancing evaluation capacity.*	Greater understanding of the evaluation process and activities to effectively support high-quality program evaluation Enhanced evaluation knowledge and skills (e.g., logic modeling, data collection and analysis, and interpreting evidence) Less fear of evaluation and more positive attitudes toward evaluation practice Enhanced evaluative thinking Modification of organizational systems, structures and processes to support high-quality evaluation

* **Source:** Patton MQ, Campbell-Patton CE. Utilization-focused evaluation. 5th ed. Thousand Oaks, CA: Sage; 2021.

^†^ **Source:** Mertens DM, Wilson AT. Program evaluation theory and practice. New York, NY: Guilford Press; 2018.

^§^ **Source:** Office of Management and Budget. Phase 4 implementation of the Foundations for Evidence-Based Policymaking Act of 2018: Program Evaluation Standards and Practice. Executive Office of the President, Office of Management and Budget; 2020:1–30.

**Evaluation types.** A clear evaluation purpose statement provides valuable insights into what type of evaluation is most appropriate to meet the specific information needs given the program’s stage of development. Several types of evaluations are possible and have been defined earlier in this report. They include, but are not limited to, formative evaluation, process or implementation evaluation, outcome evaluation, impact evaluation, and economic evaluation.

**Intended users and uses.** Users are the specific persons or groups who learn from and act on the evaluation findings. They typically are a subset of interest holders, as not all interest holders will use evaluation findings. Identifying intended users early (during Step 1) and engaging them collaboratively in conversations about what they would like to know about the program, how they intend to use this knowledge, and when they are most likely to use the findings are important considerations in focusing the evaluation.

Evaluation findings can be used in several ways (Table 4). When identifying the intended uses, items to consider include when the findings are needed, the program's stage of development (e.g., pilot phase, early implementation, or mature program), and the broader context that might affect the ability to use evaluation findings, as assessed in Step 1. All uses are linked to one or more specific users and align directly with the purpose of the evaluation. Stating uses in vague terms that appeal to many persons decreases the chances that the evaluation will fully address anyone's needs.

**Evaluation questions.** Evaluation questions tend to be broad in scope, are open-ended, establish the boundaries for the evaluation by stating what aspects of the program will be addressed, and can be answered with the data gathered from the evaluation. Evaluation questions are not the same as data collection questions (e.g., survey, interview, and focus group questions) and typically do not ask about what or how something should be done in the future.

When developing evaluation questions, engage collaboratively with interest holders to identify and prioritize the questions that they would like the evaluation to answer (Table 5). Factors that can be helpful when developing and prioritizing evaluation questions with interest holders include

**TABLE 5:** CDC Program Evaluation Framework (Step 3 — Focus the evaluation questions and design): example process and outcome evaluation questions

Process evaluation questions	Outcome evaluation questions
**Input** To what extent are the program resources sufficient for successful implementation? How do program staff qualifications align with those needed for program implementation? What gaps exist? **Activity** To what extent is the program being implemented as planned? What are the facilitators and barriers to implementation? Is the program reaching its intended audience? Why or why not? What are the overall program costs? How much does the program cost per unit of service? In what ways are or aren’t program activities designed to address the drivers of health inequities? In what ways are the program activities tailored to the community served? Where are there opportunities for improvement? What is the quality of materials provided as part of the program? To what extent are they understood across a variety of intended audiences?	To what extent is the program progressing toward its desired outcomes? Under what conditions is this program most effective and why? For whom is the program most and least effective and why? What unintended consequences (positive or negative) are associated with the program? In what ways, if any, has the program contributed to changes in policies or procedures related to the program?
**Evaluation questions with process and outcome considerations**	
To what extent were resources used efficiently to implement the program? (Inputs and activities) What is the cost-benefit of the program? How cost-effective is the program? (Inputs, activities, and outcomes) To what extent do the pathways depicted in the logic model or program roadmap align with those seen in practice? Where do the pathways differ from what was anticipated and why? (Inputs, activities, and outcomes)

- evaluation purpose,
- resources available for planning and implementing the evaluation (e.g., funding and staffing),
- timeline for conducting the evaluation,
- when interest holders need information to make decisions,
- how long the program has been in place (e.g., has it been long enough for outcomes to be achieved?),
- availability of similar insights from prior evaluations or other evidence activities, and
- whether answering the question will provide insights for advancing health equity.

The number of evaluation questions should be limited to those that can be answered in a timely manner, with the resources available, and by using appropriately rigorous methods. Formulating and prioritizing evaluation questions should establish the specific aspects of a program to be evaluated. For example, certain persons might want to understand how programs operate together as a system of interventions to effect change within a community, others might have questions concerning the performance of a single program or a local project within a program, and others might want to concentrate on specific subcomponents or processes of a project.

Clear decisions regarding what aspects of a program are within the evaluation’s scope and related to the evaluation purpose will be important in subsequent steps of the evaluation to guide method selection. The final evaluation questions should align clearly with the evaluation purpose and the intended uses of the findings while addressing the information needs of interest holders to the extent possible. Ensuring the evaluation provides insights for funders and implementers, as well as community members who might be affected by the program, is important to make sure that all perspectives are represented in the evaluation aims.

**Evaluation design.** The evaluation design provides the overarching structure for an evaluation, determining important methodological decisions (e.g., whether comparisons will be made, and if so, what types; and whether sampling will be needed, and if so, what type) (*4*). No design is better than another under all circumstances. Selection of the appropriate evaluation design is guided by multiple factors, such as the purpose of the evaluation, the evaluation questions, and the evaluation context (e.g., budget, timeline, setting, and responsiveness to interest holder needs). The selected evaluation design should be implemented in a manner that upholds the evaluation standards to the greatest extent possible. Each type of design has strengths and limitations. The choice of design has implications for what is considered evidence, how that evidence will be gathered, and what kinds of claims can be made (including the internal and external validity of conclusions).

Because evaluation is a scientific activity, evaluation design options often have drawn from other scientific disciplines such as the social and behavioral sciences (*4*). These designs are typically classified as experimental, quasi-experimental, or observational.

- **Experimental designs** use random assignment to compare the effect of a program on one group with an otherwise equivalent group that did not receive the program.
- **Quasi-experimental designs** do not use random assignment. Instead, they compare between nonequivalent groups (e.g., program participants versus those on a waiting list) or between a group at different points in time (e.g., time series).
- **Observational designs** (e.g., case studies and post-test only) are typically considered most useful for evaluations that seek answers to various noncausal questions, such as the fidelity to an original design, quality of implementation, efficiency of the program, or other general operating practices (e.g., process and implementation evaluation) (*16*).

Experimental and quasi-experimental designs are typically viewed as more rigorous design options (compared with observational) for answering questions about whether a program has contributed to or resulted in the outcomes of interest (e.g., outcome and impact evaluation) because they can often rule out other reasons why an observed outcome occurred (*73*,*74*).

As these designs have been applied in various contexts, there has been a need to develop new or modify existing approaches to accommodate nuances that often arise in evaluation contexts (e.g., the complexity of settings in which programs are implemented, the complexity of programs themselves, and contextual constraints and conditions that render certain traditional designs infeasible or inappropriate). As a result, evaluation methodologists have created and implemented various of approaches that include principles for evaluation designs that might better accommodate certain contexts (*75*). Examples include outcome harvesting (*76*), process tracing (*75*), contribution analysis (*77*), and the success case method (*78*).

Additional factors to consider when selecting an evaluation design extend beyond ensuring that the selected design aligns directly with the evaluation questions and purpose. One factor is the likelihood of implementing the design as intended. Evaluators should collaborate with interest holders who are familiar with the context to examine whether it will be possible to implement the proposed design in a manner that upholds the underlying scientific principles within the contextual constraints that exist. Examples of factors in the environment that might affect successful implementation of the design include the ability to access and engage participants successfully, the timeline for conducting the evaluation, and the resources available (e.g., funds and staff members) (*16*). Designs can only be considered rigorous when they are implemented with high quality (i.e., in alignment with underlying scientific principles).

The selected design should be culturally responsive (*28*). Interest holders might have viewpoints on the credibility of information from specific evaluation designs, which can affect whether they participate in, and act on, the evaluation findings. Evaluators need to consider how their own experiences and background might influence their design preferences. Engaging in reflective practice throughout the evaluation process can help evaluators better understand the viewpoints of others when weighing different design options and ultimately arrive at a design that produces relevant, useful, and rigorous insights in an ethical manner.

**Implementation considerations.** When implementing an evaluation, the context or data needs might change. For example, the intended use for the evaluation might shift from improving a program’s current activities to determining whether to expand program services to a new population group (*36*). Interest holders and other persons involved in the process might find that something was overlooked within the context during the planning phase that makes it difficult or impossible to continue with the design as planned and still achieve the evaluation aims (e.g., envisioned participants are no longer available). These types of shifts in the environment require that evaluators are adaptive in their approach (*36*) and might result in a need to revisit and modify the evaluation design, evaluation questions, and even the purpose.

Certain decisions about the evaluation design can make it more challenging to adjust once the evaluation is in the implementation stage (e.g., the design includes multiple intervention and comparison sites that already have been approved through ethics reviews). These challenges do not indicate that certain designs are to be avoided, rather they emphasize the importance of ensuring that thorough discussions are held during the planning phase with careful consideration to the feasibility of implementing the selected design (Table 6).

**TABLE 6:** CDC Program Evaluation Framework (Step 3 — Focus the evaluation questions and design): example questions to consider when applying cross-cutting actions and evaluation standards

Example question	Cross-cutting action		
	Engage collaboratively	Advance equity	Learn from and use insights
Were interest holders engaged in the development, identification, and prioritization of evaluation questions? Are evaluation question(s) included that will provide insights for advancing health equity?	Relevant	Relevant	Relevant
Were interest holders who are familiar with the context engaged during the design process, to ensure it will be possible to implement the proposed design? Is the selected evaluation design culturally responsive and viewed as credible by interest holders?	Relevant	Relevant	—
Did evaluators engage in reflective practice throughout the evaluation design process to better understand the viewpoints of others when weighing different evaluation design options to produce relevant, useful, and rigorous insights in an ethical manner?	—	Relevant	Relevant
Will the final set of evaluation questions address information needs of various interest holders including community members who might be affected by the program? Is information included about how the findings are likely to be used, at what points in time, and who is likely to learn from or use the findings?	—	—	Relevant
How to apply the evaluation standard	Evaluation standard		
Collaborate with interest holders on focusing the evaluation questions and design to increase the likelihood they will find the evaluation results relevant to their specific situation and responsive to their needs. The resulting evaluation findings will be more relevant and useful to interest holders, increasing the likelihood they will take action on the findings.	Relevance and utility		
Develop a clear purpose statement and high-quality evaluation questions to provide a clear aim for the evaluation that can be addressed within the time frame and budget. Intentionally considering which of several design options will directly address the evaluation questions, are feasible to implement, and are aligned with the context increases the likelihood that the selected design will be carried out in a rigorous manner.	Rigor		
Collaborate with interest holders in establishing the evaluation purpose and identifying and prioritizing evaluation questions to decrease the likelihood that questions will be selected that are important only to interest holders who hold the most power (e.g., funders). This action can increase the objectivity of the process.	Independence and objectivity		
Document the evaluation purpose and questions to make clear to interest holders what is within the scope of the evaluation and why. Such transparency can be helpful if new interest holders become engaged after the evaluation is underway, particularly if they have different or competing interests.	Transparency		
Consider that when developing an evaluation design, decisions are often made about who will contribute data for the evaluation and how they will participate. Such decisions can naturally lead to including some and excluding others, and raise questions regarding ethics. Engaging in intentional discussions with interest holders, particularly persons who are aware of the context, can help identify potential ethical concerns and identify means for addressing them.	Ethics		

#### Step 4: Gather Credible Evidence

This step builds on the high-level evaluation design (evaluation purpose, evaluation questions, and design) developed in Step 3 to determine the evidence needed to answer the evaluation questions, including what data will be collected, how, when, and from who or what. The product of this step includes a data collection strategy that defines expectations for credible evidence, methods that will be used to ensure data quality, indicators and associated measures of interest, and data sources. Evaluators collaboratively engage with interest holders to consider the credibility of the approaches and the rigor of resulting data and decide what specific data to collect, and how, in response to these information needs (Table 7) (Table 8).

**TABLE 7:** CDC Program Evaluation Framework (Step 4 — Gather credible evidence): example of alignment of evaluation question, design, construct, indicator, measure or metric, and expectation

**Evaluation question:** To what extent did exposure to the community campaign increase knowledge of the health risks of smoking among smokers?				
**Evaluation design:** A pre/post design in which a community receives a campaign over a 3–6 month period, establishing statistical comparisons between campaign on and off-air periods.				
Construct or concept	Indicator	Measure or metric	Data type and source	Expectation
Knowledge of health risks of smoking	Level of confirmed awareness of media messages on cigarette smoking and health conditions	Difference in % of respondents who believe cigarette smoking is related to specific health conditions	Data type: online survey* Source: designated media market community members	A statistically significant higher knowledge of campaign specific messages in the community when the campaign is on air than when the campaign is off air^†^
	Level of confirmed awareness of media messages that smoking can worsen medical complications from diabetes	Difference in % of respondents who agree it is highly likely that smoking will worsen medical complications from diabetes		
	Level of confirmed awareness of media messages that smoking can result in immediate damage to the body	Difference in % of respondents who agree that smoking can cause immediate damage to the body		
Awareness of campaign	Level of self-reported exposure to campaign	Difference in % of respondents who report exposure to campaign messaging	Data type: online survey* Source: designated media market community members	A statistically significant higher knowledge of campaign specific messages in the community when the campaign is on air, than when the campaign is off air^†^

* Surveys are administered before and 1 week after the campaign has started. Difference scores are preimplementation scores that are subtracted from the 1-week scores.

^†^ Knowledge differences might vary depending on baseline data, duration of the campaign (i.e., recommended minimum of 3–6 months), dose level, and the media mix.

**TABLE 8:** CDC Program Evaluation Framework (Step 4 — Gather credible evidence): example questions to consider when applying cross-cutting actions and evaluation standards

Example question	Cross-cutting action		
	Engage collaboratively	Advance equity	Learn from and use insights
Does the data collection approach include multiple data sources and incorporate different perspectives to provide a comprehensive view of the program? Is the data collection approach reflective of what interest holders view as credible evidence?	Relevant	Relevant	Relevant
Have interest holders been engaged to discuss the data collection approach, which data to collect, how to collect it, identify opportunities to reduce data collection burden, and streamline data collection? Have there been discussions with interest holders regarding data ownership and data sovereignty? Have the evaluators considered how their professional and lived experiences affect their preferences for what constitutes credible evidence?	Relevant	Relevant	—
How to apply the evaluation standard	Evaluation standard		
Collaborate with interest holders to better understand the types of data and data sources they would find most credible for answering the evaluation questions to increase the likelihood that they will make use of the findings. Clearly connecting the evaluation questions and purpose statement in Step 3 with the data collection strategy (i.e., data collection methods, concepts and constructs, indicators, and measures or metrics) developed in Step 4 increases the likelihood that the data will answer the evaluation questions identified with interest holders as relevant.	Relevance and utility		
Discuss with interest holders which data collection options are feasible to implement in the context and best align with the cultural norms to increase the likelihood of participation from potential respondents, improving validity and rigor. Developing and reviewing collection instruments with interest holders familiar with potential respondent groups can help to appropriately tailor wording, thereby increasing the likelihood that respondents will better understand what is being asked and as a result provide more accurate responses.	Rigor		
Collaborate with various interest holders in designing a data collection strategy to ensure that multiple perspectives about what constitutes credible evidence are incorporated. This increases the likelihood that the data collection strategies will reflect broad input beyond those who might traditionally have the most influence on the evaluation design (e.g., funders).	Independence and objectivity		
Document how evaluation data gathered align with the evaluation questions to provide a foundation for explaining how the findings and recommendations are supported by the data. Sharing, discussing, and documenting expectations about what results are suggestive of the extent to which a program is doing well make it clear what will be taken into account when interpreting the findings. When expectations cannot be set in advance, documenting the procedures that will be undertaken to interpret the findings in Step 5 (e.g., who will be involved, envisioned structure, and content of discussions) can also provide transparency.*	Transparency		
Ensure and document how persons providing data for the evaluation will be made aware of any associated risks; what will happen with the data they provide; how privacy and confidentiality will be upheld in the process; and how the data will be handled, stored, and used is essential to protecting the rights of those who participate. Discussing how to structure evaluation efforts so they do not cause or create harm within the community is an essential component of ethical practice. Having a clear plan for how the time and knowledge participants contribute will be recognized is an important form of reciprocity. It is important to be aware of and adhere to the specific rights, rules, and procedures within different contexts relating to data collection, ownership, and sharing of knowledge and insights (e.g., tribal data sovereignty).^†^	Ethics		

* **Source:** Greene JC, Boyce AS, Ahn J. Value-engaged, educative evaluation guidebook. Urbana-Champaign, IL: University of Illinois at Urbana-Champaign; 2011. https://comm.eval.org/viewdocument/eval11-session-316

^†^ **Source:** Eakins D, Gaffney A, Marum C, Wangmo T, Parker M, Magarati M. Indigenous evaluation toolkit for tribal public health programs: an actionable guide for organizations serving American Indian/Alaska Native communities through opioid prevention programming. Seattle, WA: Seven Directions; 2023. https://assets-global.website-files.com/5d4b3177c03a6439be501a14/63f550f6aca5a76fe89c290a_FINAL_7D_EvalToolKit_FullDoc_022123_WEB_compressed.pdf

**Establish expectations.** Establishing expectations involves evaluators and interest holders engaging collaboratively to determine what evidence will be used to answer the evaluation questions; what expectations they have about the type, quality, and quantity of data needed; and what changes, trends, or patterns suggest the program is on track or doing well. These discussions might include the types of evidence that are most valued by different groups (e.g., quantitative and qualitative) and the perceived credibility of data sources. In an outcome evaluation, this also might include discussing which outcomes will be examined and identifying the accountable outcome (i.e., the most distal outcome interest holders expect the program to show progress toward achieving). Establishing these expectations is critical before determining the methods and measures to use in answering the evaluation questions.

Another type of expectation relates to understanding what type and level of results will be used to answer the evaluation questions. For example, an evaluation inquiring about a program’s efficiency will need to demonstrate an understanding of what level of efficiency is considered excellent, good, adequate, or poor. Explicitly identifying these expectations improves transparency and provides a point of reference with which to compare results and see if expectations were met. Various methods can be used to identify these expectations. For example, evaluators and interest holders could examine previous patterns in program data, consult the literature for research and evaluation studies conducted on the same or similar topics, review industry standards, or consult with interest holders who might offer their own perspectives regarding these expectations. Discussing, understanding, and coming to consensus on these expectations can facilitate the use of evaluation findings and create transparency around how the evaluation findings will be interpreted (*43*).

Cultural norms might vary regarding what constitutes credible evidence (*79*,*80*). Understanding these norms, respecting different vantage points, and collaboratively engaging with interest holders to identify how to collect data in a way that is meaningful and useful in that context are important for conducting a high-quality evaluation (*13*). In addition, interest holders might have different perspectives regarding what constitutes rigorous and credible data. When discussing how to answer the evaluation questions, certain interest holders might value quantitative data and associated statistics, whereas other interest holders might value narratives from qualitative data. An evaluation should strive to collect data that will convey a well-rounded picture of the program in a manner that is rigorous and credible for interest holders. When interest holders are involved in discussing and defining data that will be credible in their contexts, they will be more likely to trust the results and be more invested in the evaluation’s conclusions and recommendations, which will enhance the likelihood that findings will be acted on (Step 6).

**Methods.** The overall evaluation design was identified in Step 3. During this step, evaluators and interest holders will make decisions about how to gather evaluation data. Each data collection method has strengths and limitations, and no data collection method provides a complete picture. Evaluators and interest holders will need to weigh the pros and cons of methods to arrive at the best approach for the evaluation in the specific context taking into account any associated constraints. Consulting statistics experts might be necessary, such as in situations where there is a need for methods or types of data to make inferences about the program’s success.

Describing the many quantitative (i.e., numeric) or qualitative (i.e., narrative) data collection methods available (e.g., surveys, interviews, focus groups, observations, document and record reviews, and journals or diaries) is beyond the scope of this report. Although quantitative and qualitative methods are often implemented separately, a mixed methods approach in which the two are purposefully integrated can improve the accuracy of the results by compensating for limitations in one method with another that is strong in that area or acquiring deeper insights on a topic to improve understanding (e.g., explaining the “why” behind quantitative survey results through follow-up interviews) (*43*,*81*).

Ensuring that the evaluation data collection methods are culturally responsive is fundamental to ensuring trustworthy and accurate data (*82*). Various considerations exist when adapting or developing instruments, such as appropriately matching the instrument type to the context (e.g., not using a written survey in an oral-based culture), ensuring the appropriate reading level is used for written instruments, taking steps to confirm that translations of instruments into different languages are accurate, and allowing respondents to share information in their preferred language (*28*,*83*,*84*).

Although new data might need to be collected to answer the evaluation questions, before committing to gathering new data, evaluators and interest holders might explore whether there are data already available that might be able to answer some or all evaluation questions. For example, public health surveillance, education, census, or other large data sets might be available and analyzed at a much lower cost than primary data collection. These data sources might not perfectly align with the ideal data or participant group, but if they align well with the evaluation question of interest and are trustworthy, the tradeoff might be worth using data that do not involve additional expenditures.

**Indicators.** Indicators are measurable statements and serve as a bridge between general program constructs or concepts and specific metrics or measures that can be interpreted (*70*,*74*,*85*,*86*). For example, a construct of social connectedness might have an indicator of the quality of relationships that could be measured by the percent of persons in a community who report having a close bond with at least one person. Checking whether there are metrics with sufficient specificity and detail for accurate data collection is important. The program roadmap or logic model, when used in conjunction with the evaluation questions, is useful for guiding development and decisions related to indicators, which can relate to any part of the program. Common indicator categories include the following:

- **Inputs.** The resources needed for conducting program activities (e.g., personnel, materials, funding, equipment, surveillance data, partnerships, and existing evidence base).
- **Activities.** Characteristics or qualities of the program implementation efforts (e.g., screening for disease, delivering an immunization campaign, or conducting training). This category includes outputs, which are the products of program activities (e.g., children screened who meet risk profile, vaccinations administered, or community members who completed training using culturally appropriate curriculum).
- **Outcomes.** The expected program effects or changes in the short, intermediate, and long term (e.g., increased antitobacco attitudes, increased intentions to exercise, increased immunization rates, and decrease in morbidity and mortality due to breast cancer).

Outcomes can be more challenging to measure than inputs or outputs. In addition, long-term outcomes are often difficult to attribute to a single program, because usually, multiple factors contribute to changes in outcomes. As a result, it is sometimes tempting to focus evaluation questions and the indicators that align with these on inputs, activities, and outputs. However, programs are encouraged to measure outcomes rather than just outputs (if appropriate for the stage of the program and evaluation questions).

To advance equity, it is important to consider whether the proposed indicators will provide valuable information about the drivers of health inequities. Indicators, and the measures associated with them, not only provide insights about what or how much of something is happening, but also for whom and under what conditions. Logic models and discussions with interest holders can provide helpful ideas for indicators to use in responding to the evaluation questions.

Measures associated with indicators might be quantitative or qualitative, depending on the evaluation question being answered. For example, understanding why a training did not achieve increases in knowledge or changes in attitudes might be best captured through qualitative data, whereas changes in knowledge might be captured using quantitative measures.

Multiple indicators are often needed for responding to the evaluation questions; however, too many indicators can detract from the evaluation goals, take valuable resources to collect and analyze, and be burdensome for persons or organizations providing the data. Thus, evaluators and interest holders might want to consider that certain indicators will be more time-consuming and costly than others to measure and carefully consider the level of effort associated with each before making a final decision on which to include.

**Data sources.** Data might be provided from various sources and might include new data collected specifically for the evaluation (primary data) or existing data (secondary data that might be available within the program being evaluated or in external organizations). If possible, using multiple sources provides an opportunity to include different modes and perspectives, potentially enhancing the evaluation’s rigor and credibility. For example, a perspective from inside a program could come from internal documents and interviews with staff or program managers, whereas clients and persons with lived experience, neutral observers, or those who do not support the program might provide a different but equally relevant perspective. Considering these and other perspectives provides a more comprehensive view of the program.

A key decision point related to data sources is whether information needed to answer evaluation questions will be collected from all units of a specific source (e.g., all recipient reports submitted) or a subset (e.g., random sample of all recipient reports). If sampling is needed, the criteria used and rationale for the sampling strategy should be stated clearly to provide information that interest holders can use to interpret the evidence accurately and assess potential biases (*16*).

**Data quantity and quality**. When collecting data, consider the quantity needed. Collecting the appropriate amount and types of data to answer the evaluation questions sufficiently (i.e., need to know) is important, as is avoiding the desire to collect data that might be tangential to answering evaluation questions (i.e., nice to know). Balancing the amount of data with the burden (in terms of time commitment and effort) data collection can place on the respondents and others who might be involved in data collection and processing can be challenging. Collaboratively engaging with communities can help ensure the right balance is struck for the specific context.

Data quality refers to the appropriateness and integrity of the data used in an evaluation (*58*). High-quality data are reliable, valid, authentic, and informative for their intended use. Well-defined indicators enable easier collection of quality data because they clarify what specific data are viewed as credible and necessary to answer the evaluation questions. Other factors affecting quality include instrument design, data collection procedures, training of data collectors, source selection, coding, data management, data cleaning, and error checking. Obtaining quality data will entail tradeoffs (e.g., breadth versus depth), and discussing the options with interest holders when planning the evaluation can highlight how certain tradeoffs might affect perceived data credibility.

**Data collection and context considerations.** The timing and infrastructure for collecting, handling, and storing data, and the cultural context need to be considered when making decisions regarding gathering data. Persons providing data should be knowledgeable about their rights; any associated risks; and how the data will be handled, stored, and used, including how privacy and confidentiality will be protected in the process (*16*).

Persons and organizations might have cultural norms regarding appropriate permissions to engage in data collection, identifying who will collect the data, data governance processes, and acceptable ways of asking questions and collecting data (*42*). For example, certain participants might be willing to discuss their health behaviors with a stranger, whereas others might be more at ease with someone they know. Working with interest holders on the evaluation data collection procedures can help ensure they align with the project setting, that privacy and confidentiality are protected, and ethical practices are upheld (*16*).

Persons who provide data for the evaluation contribute time, energy, and knowledge. Without their involvement, evaluations would not be possible. In developing a data collection plan, discuss how persons will be acknowledged for their important contributions (*38*). Collecting data that are not extractive is important, and evaluators need to work with the project team to share data with communities and respect data sovereignty.

**Implementation considerations.** Despite the best planning, data collection challenges are common once an evaluation has commenced. For example, even though evaluators planned for and addressed data concerns in advance, once the evaluation starts, persons or organizations might have unease and concern about providing access to data sources or might answer questions in a more favorable light than truly represents a situation for fear that the evaluation will show inadequacies in the program and consequences will result (e.g., less funding and program cancellation). Such reactions are demonstrations of evaluation anxiety (*87*). Engaging collaboratively with those invested in or affected by the evaluation early in the evaluation planning process, and often throughout the evaluation implementation, can help to establish trusting relationships and reduce concerns that might stem from the unknown.

Data collection plans might change once implementation begins. Access to certain data might not be available as originally anticipated, response rates might be lower than necessary, and information needs might change (e.g., evaluation findings are needed sooner than originally expected because of a change in circumstance) requiring a modification to the data collection approach. Such changes need to be documented throughout an evaluation so that persons making use of the findings can make a well-informed decision about the quality and trustworthiness of the work performed. Documenting modifications provides a level of transparency required of high-quality evaluations (*13*,*16*,*26*).

#### Step 5: Generate and Support Conclusions

Activities in Step 5 focus on generating answers to the evaluation questions (Table 9). These answers are presented as evaluation conclusions, align with the evaluation questions (Step 3), and demonstrate how the conclusions are supported by the data collected (Step 4). This step involves reviewing the evidence expectations identified previously, conducting robust data analysis, interpreting findings, and developing recommendations. Engaging interest holders in the process of interpreting analyses, drawing evaluative conclusions, and testing the feasibility of potential recommendations will help ensure the conclusions and suggested actions are tied to the underlying data and responsive to the context.

**TABLE 9:** CDC Program Evaluation Framework (Step 5 — Generate and support conclusions): example questions to consider when applying cross-cutting actions and evaluation standards

Example question	Cross-cutting action		
	Engage collaboratively	Advance equity	Learn from and use insights
Have specific conversations taken place to identify opportunities for incorporating analyses that might contribute valuable insights for advancing health equity?	Relevant	Relevant	—
Has time been allocated for the evaluators and interest holders to work together to interpret data analysis results within the context and to translate what the findings mean? Have efforts been made to support evaluation recommendations that are actionable and feasible to implement in the context?	Relevant	—	Relevant
How to apply the evaluation standard	Evaluation standard		
Collaborate with interest holders to interpret analyses to increase understanding of the findings and greater receptivity and commitment to learning from and using the findings. Engaging with interest holders to better understand the likelihood of acting on potential recommendations and providing a focused set of recommendations can improve relevance and usefulness.	Relevance and utility		
Establish a plan for how the data will be analyzed in advance of data collection to help ensure that any new data collected include questions necessary to produce measures aligned with the indicators established in Step 4 and the evaluation questions identified in Step 3. Acquiring data from multiple sources and using multiple methods can lead to more rigorous findings by providing a more complete picture of the issue being evaluated.	Rigor		
Interpret the evaluation findings by comparing analytic results to a set of expectations that was cocreated with interest holders in Step 4 to help increase the likelihood that multiple voices and perspectives, as well as identified biases, are considered when interpreting findings.	Independence and objectivity		
Document the strengths and limitations of the evaluation to provide valuable information for interest holders who make decisions about whether to act on the evaluation recommendations. In addition, interpreting the analytic findings by comparing the results to the expectations established in Step 4 provides clarity for those wanting to learn from and use the findings about how the recommendations were derived from the evaluation data gathered.	Transparency		
Ensure evaluators contribute to upholding the ethics standard by presenting analytic findings in a manner that protects the privacy and confidentiality of persons and entities that provided data (e.g., ensuring information that could identify a person is not shared).	Ethics		

**Analysis.** The plan for analyzing evaluation data should be established in advance of implementing the evaluation and use the most robust methods possible to answer the evaluation questions. Developing an analysis plan before data collection will increase the likelihood that data collection instruments include questions necessary to acquire the data needed to produce measures aligned with the indicators established in Step 4.

Whether conducting an analysis of quantitative, qualitative, or both types of data, each type of analysis has established procedures for upholding rigor and objectivity and considerations for protecting privacy and confidentiality that should be followed. Identifying and describing the multitude of analytic methods available is beyond the scope of this framework. Regardless, decisions about which analytic approach(es) to use need to be guided by the evaluation questions and characteristics of the data collected. As noted in Step 4, involving statistics experts might be necessary for analyses and interpretation, particularly for complex analyses, as incorrect or inappropriate analysis or interpretation can lead to false claims and potentially result in decreased trust among interest holders.

The decisions in Step 4 regarding measures and sources will inform the analysis plan and, if there are multiple measures or data sources to answer evaluation questions, describe how to synthesize across them. Having multiple sources and methods can help in interpretation, drawing conclusions, and making recommendations because they provide more information to learn from than singular data sources.

Engaging collaboratively with interest holders regarding the types of analysis that will be needed is important. Certain evaluation questions might be addressed through descriptive analyses, whereas others might require more advanced analyses. Discussions with interest holders also should include how to incorporate analyses that might contribute valuable insights for advancing health equity.

**Interpretation.** Simply reporting analytic results is insufficient to draw evaluative conclusions. Results of data analyses are compared with the expectations identified earlier (Step 4) and interpreted within context (Step 1) to determine the practical application and implications of what has been learned. In Step 5, evaluators and interest holders work together to translate what the findings mean, identifying existing strengths, successes, and areas for improvement including opportunities to advance health equity (Box 2). Engaging collaboratively to interpret the findings has multiple benefits including producing a more robust understanding of the findings and their implications and enhancing interest holders’ receptivity and commitment to learning from and using the evaluation findings.

BOX 2 **2024 CDC Program Evaluation Framework (Step 5 — Generate and support conclusions): considerations for involving interest holders when interpreting analytic results**

**Table:** 

**Planning for interpretation of results with interest holders** Establish a process for interpretation that includes interest holders. Consider the resources and capacity needed to engage interest holders in reviewing analytic results and to make data and methods accessible and relevant (e.g., plain language and data literacy). Ensure the evaluator or facilitator has a thorough knowledge of the context and interest holders. Include various culturally and contextually responsive formats for presenting results (e.g., tables, charts, pictures, quotes, stories, and maps). Consult with interest holders to determine a convenient time for them to participate, and a setting that is convenient and comfortable for open discussion. **During the collaborative interpretation process** Provide transparency around the choices made on methodology, data collection questions, and the data analyses conducted, which shape the results produced. Facilitate openness to incorporating new or differing points of view and valuing various sources of expertise. Consider various types of engagement in the interpretation process, allowing individual or group input and being sensitive to group size or dynamics. Discuss how the interpretation is situated in context, who the results and findings are relevant for, and the various points of view that can be included and emphasized in the narrative. Determine the level of certainty around the interpretation. Discuss and identify who will be acting on the findings and recommendations. Develop recommendations collaboratively based on the results.

**Source:** Krause, H., and Richburg-Hayes, L. The Data Equity Framework: a concrete and systematic equity-oriented approach to quantitative data projects; 2023. https://doi.org/10.31235/osf.io/sqt4u

Where an existing evidence base exists, evaluation conclusions can be further strengthened by interpreting the analytic findings within the context of this evidence base. Furthermore, scientific theories or models (e.g., theory of planned behavior or diffusion of innovation) identified in earlier steps or in existing literature also might be used to explain findings.

**Recommendations.** Recommendations are actions for consideration resulting from the evaluation and can suggest how improvements could be made and how existing successes and strengths can be leveraged (*88*). Similar to the evaluative conclusions formed when interpreting findings, recommendations also are rooted in the evaluation findings and need to be supported by the evidence.

When formulating recommendations, the broader framework steps should be considered, particularly Step 6 (Act on findings). Using insights to create actionable recommendations is critical to creating meaningful program changes. Recommendations are more actionable when they are clearly worded, provide multiple potential options for action, are in alignment with potential users’ roles and responsibilities, and are feasible to implement in context (i.e., can be implemented within fiscal, time, staff, and other constraints) (*88*).

Engaging collaboratively with persons who are aware of the context and potential constraints and opportunities within the environment(s) in which recommendations will be implemented can be done while formulating potential recommendations so evaluators can learn about the potential feasibility of acting on them and adjust accordingly. To further increase the likelihood of facilitating action, evaluators might consider limiting the number of recommendations and prioritizing them (*88*) and providing suggestions for who could be responsible for taking action, on what timeline, and how the implementation of actions might be monitored (if known and appropriate for the context).

**FIGURE 3:**
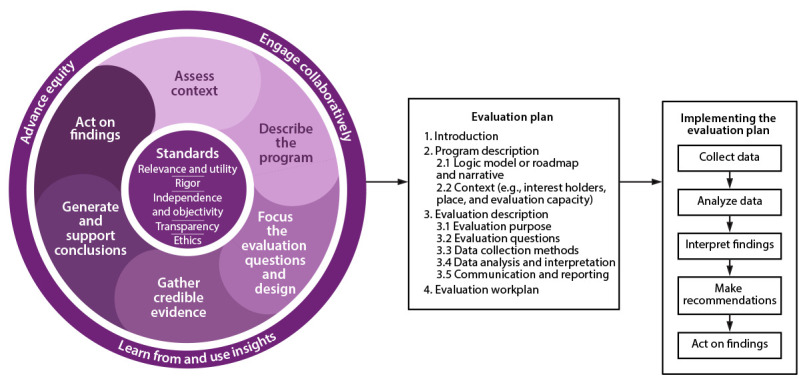
Sequence of the CDC Program Evaluation Framework informing development* and implementation^†^ of an evaluation plan to generate and support conclusions * Example evaluation plan outline; might differ depending on program evaluation and context. ^†^ Continued application of cross-cutting actions and evaluation standards. Ongoing consideration of culture and awareness of and adaptation to changing context.

**Implementation considerations.** All evaluations have strengths and limitations. Ensuring that both are articulated alongside the analysis approaches, interpretations, and recommendations is important for transparency. Much of the time spent on an evaluation is often allocated to earlier steps in the framework. Implementation of an evaluation plan in large part includes interpreting and understanding the findings resulting from data collection and analysis and using that information for recommendations and acting on findings (Figure 3). It is important that sufficient time is allocated for synthesis and working with interest holders on interpretation and recommendations (*43*).

When working with interest holders who might not be as familiar with analysis and interpretation methods, it might be helpful to engage in ways that clearly and plainly summarize the procedures and findings, including strengths and limitations, to ensure the connection between findings and data are transparent and clear. This is also an opportunity for a collaborative approach to understanding and interpreting the meanings of the findings and to hear from interest holders who might have a different perspective or interpretation. Understanding and incorporating these perspectives into the products will improve the likelihood that the results and recommendations will accurately represent the context and be accepted and used by interest holders.

#### Step 6: Act on Findings

Evaluation findings, recommendations, and lessons learned are crucial for improving programs; however, they do not automatically translate into action for informed decision-making. Using evaluation data and insights remains an elusive goal for many organizations. To ensure evaluation insights are used requires early planning, collaboration, and commitment from the evaluator and all interest holders to act on the findings and recommendations. This step is an essential element in the evaluation cycle and is important not to overlook (Table 10). Key elements for acting on the findings of an evaluation include planning, preparing findings for use, and facilitating insights to action.

**TABLE 10:** CDC Program Evaluation Framework (Step 6 — Act on findings): example questions to consider when applying cross-cutting actions and evaluation standards

Example question	Cross-cutting action		
	Engage collaboratively	Advance equity	Learn from and use insights
In what ways have interest holders been engaged to discuss how they plan to use the findings and recommendations?	Relevant	—	Relevant
How are regular communication and feedback opportunities with interest holders being planned throughout each step of the evaluation process?	Relevant	—	Relevant
How can the evaluation team support interest holders in acting on the findings after the results are disseminated? In what ways can the team facilitate and engage interest holders in using evaluation findings to inform decision-making?	Relevant	—	Relevant
How have communication preferences and cultural norms been taken into consideration in the planning for dissemination and reporting of findings?	—	Relevant	Relevant
How to apply the evaluation standard	Evaluation standard		
Create a well-crafted plan for who will use the findings, when, and how to facilitate learning and use to help make the findings relevant and translate them into action. Considering and acting upon opportunities that exist to facilitate decision-making conversations using evaluation insights throughout the evaluation process can also increase the use of findings. To increase the likelihood of use, evaluators can prepare interest holders in advance for using the evidence by discussing how potential findings (positive and negative) might impact decision making.	Relevance and utility		
Ensure evaluators work with interest holders to prevent the misuse of findings, including over emphasizing negative or positive findings or taking results out of context to use them for purposes other than those agreed upon.	Rigor		
Use multiple forums that align well with the context and include various interest holders to facilitate learning and reduce bias in interpretation and application.	Independence and objectivity		
Engage in discussions throughout the evaluation about interim findings and the status of the evaluation activities, rather than only engaging in conversations about evaluation findings at the end, to contribute to upholding the transparency standard and help to minimize the likelihood that findings will take interest holders by surprise.	Transparency		
Ensure evaluators contribute to upholding the ethics standard by ensuring that the results of the evaluation are shared back with those who provided data.	Ethics		

**Planning.** Much of the planning for acting on the findings and recommendations has been discussed in previous steps of the framework. For example, in Steps 3 and 4, collaborative engagement with interest holders about evaluation questions, methods, and indicators has included how they plan to use the findings and recommendations.

Evaluation planning needs to begin with the end in mind (*89*), asking questions about who will use the evaluation insights, what their needs are, how and when they intend to use the evaluation insights, what potential uses exist (beyond those already anticipated), and how to best facilitate and promote the use of findings and recommendations (*36*).

A well-crafted strategy that lays out a plan for who will use the findings, when, and how to facilitate learning and use is beneficial for facilitating action (*48*). Furthermore, the process of creating this strategy will highlight how interest holders can enhance the relevance, credibility, and overall utility of the evaluation. When designing this strategy, consider who could learn from the findings, how the findings can be provided quickly and often, what ways of presenting the insights will resonate most given the context, and what opportunities exist to facilitate decision-making conversations using evaluation insights.

**Preparing findings for use.** Preparing findings for use refers to the steps needed to ready the insights and recommendations for dissemination and action. Various methodologies and frameworks offer evaluators a structured approach to move from the evaluation findings to learning and use (e.g., knowledge to action and data to action) (*90*–*92*), including guiding users through how potential findings (including negative findings) might affect decision-making. This can prepare interest holders for eventually using the evidence and make space for identifying options for program improvement. Evaluators can consider ways to prime users for uptake and learning by considering issues such as how users will receive and understand the findings; how users can apply the insights in their work; and how the evaluator can help persons to use and understand the findings.

Preparation also can include various ways to disseminate evaluation findings to all interest holders in a timely, unbiased, and consistent fashion (Box 3). In addition, well-designed plans include opportunities for dialogue about how to use the insights and implement recommendations. Interest holder communication and feedback are an integral part of evaluation, particularly for learning from and using evaluation findings.

BOX 3 **CDC Program Evaluation Framework (Step 6 — Act on findings): potential methods for sharing interim and final results**

**Table:** 

Reports and briefs: provide information on an evaluation, including purpose, methods, insights or findings, and recommendations. Data dashboards, visualizations, and infographics: visual displays of key findings that can be updated, shared, and discussed with interest holders throughout the evaluation process (**Source:** Smith VS. Data dashboard as evaluation and research communication tool. New Dir Eval 2013;140:21–45). Collaborative discussions of analytic results (e.g., data walks [**Source:** Murray B, Falkenburger E, Saxena P. Data walks: an innovative way to share data with communities. Washington, DC: Urban Institute; 2015. https://www.urban.org/sites/default/files/publication/72906/2000510-Data-Walks-An-Innovative-Way-to-Share-Data-with-Communities.pdf], data gallery walks, data parties, and data chats [**Source:** Cohen M, Rohan A, Pritchard K, Pettit KLS. Guide to data chats: convening community conversations about data. Washington, DC: Urban Institute; 2022. https://www.urban.org/research/publication/guide-data-chats-convening-community-conversations-about-data]): coordinated events where interest holders convene to collaboratively discuss and make meaning of analytic results and implications for practice. Panels and townhalls: presentation of the evaluation plan, findings, or recommendations, with emphasis on gathering interest holder input, discussion, and answering audience questions. Conference presentations, webinars, and roundtables: presentation of the findings and recommendations by a speaker in either a discussion-focused format or a traditional lecture-style format, used as a way to share project findings and seek audience feedback. Papers and publications: formal dissemination as white papers or in peer-reviewed journals that describe the overall evaluation plan and process including background, process, methods, findings, and recommendations. Project meetings: regularly scheduled meetings to discuss findings and how to use findings throughout the evaluation process. Websites, social media, and newsletters: brief, high-level sharing of the findings and recommendations shared on websites, through social media, or via email. Blogs: narrative information shared on a website that allows flexibility in the scope of what is shared (e.g., broad or specific) while also providing readers with the option to leave feedback. Visual recordings and geographic information systems or story maps: drawings, illustrations, or photos that visually depict main findings. Can be accompanied by text. Storytelling (**Source:** Eakins D, Gaffney A, Marum C, Wangmo T, Parker M, Magarati M. Indigenous evaluation toolkit for tribal public health programs: an actionable guide for organizations serving American Indian/Alaska Native communities through opioid prevention programming. Seattle, WA: Seven Directions; 2023. https://cdn.prod.website-files.com/5d68735d677c2aa989f0317b/667d8a485241a86e293394be_7D_EvalToolKit_FullDoc_061124_WEB.pdf), podcasting, or photo or visual sharing: visuals or a plot, title, character, scenery, challenge, and resolution to share evaluation findings by using the voice, mind, and connection to inspire feedback and engagement. Media outreach or news release: verbal or written communication about the main findings.

Dissemination is not the final act of the evaluation; it is a cycle that evaluators conduct regularly. Dissemination can take multiple forms at each stage of an evaluation. During the evaluation, these include in-process data sharing, user check-ins, and feedback sessions with interest holders. Sharing findings early and often, even when data analysis is still in-process, and seeking input from users create an atmosphere of trust. This communication also can keep an evaluation on course by keeping those involved informed regarding how the evaluation is proceeding and how to make ongoing adjustments to the program. Evaluators can hold periodic discussions during each step of the evaluation process and routinely share interim findings, provisional interpretations, draft highlights, lessons learned, and promising practices (*89*).

Although evaluation documentation is needed, a formal evaluation report is often not the most critical product (*93*,*94*). As with other evaluation elements, the reporting strategy can be discussed in advance with intended users and other interest holders. Such consultation increases the likelihood that the information needs of relevant audiences will be met. Planning effective communications requires that evaluators consider the timing, style, tone, message source, vehicle, and format of information products. Items to consider specifically when developing evaluation products include tailoring the content for the audience; explaining the program roadmap and focus of the evaluation; and sharing data methods and findings, recommendations, and the evaluation strengths and limitations. Ensuring the use of simple, culturally responsive, and effective data visualization techniques is also important (*95*–*98*). Materials need to be clear and communicated in plain language that can be understood by the intended audience(s). Furthermore, the dissemination format, content, and language used should be informed by and responsive to the context and audience and adhere to principles of equitable communication (*19*,*20*,*99*).

**Facilitating insights into action.** Evaluators take on the role of facilitators when they commit to seeing the evaluation insights used (*49*). Evaluators help to make sense of and interpret the findings, uncover and apply insights, encourage learning, and lead groups to see ways they can be used to improve the program. Users require support from evaluators when they receive evaluation findings, with each discussion of evaluation findings offering an opportunity for users to engage with the insights. Active facilitation is necessary to guide groups to understand and use the insights as well as new uses that might emerge. Follow-up also might be required to prevent lessons learned from being lost or ignored in the process of making complex or sensitive decisions. To guard against such oversight, the evaluator serves as an advocate for the evaluation findings during the decision-making phase, facilitating understanding of what was discovered and what actions are consistent with the findings.

Facilitating the use of evaluation findings also includes preventing misuse (*90*). Evaluation results are always bound by the context in which the evaluation was conducted. However, certain interest holders might be tempted to take results out of context or to use them for purposes other than those agreed on (*100*,*101*). An example of misinterpretation of results is intentionally selecting certain results that do not reflect the overall analyses and interpretation of the evaluation. Those seeking to undermine a program might misuse results by overemphasizing negative findings without considering the program’s positive results, which is a violation of scientific integrity (*81*). Evaluators can work to prevent misinterpretations and misuse by collaboratively engaging interest holders who intend to use the findings throughout the evaluation process (*101*), ensuring that evidence is well-understood and that it is not applied to questions other than those in the evaluation, and that the findings are shared holistically rather than picked to support a particular point of view.

**Implementation considerations.** Evaluators might have additional opportunities to share information about the evaluation throughout the implementation process as opportunities arise. For situational awareness, evaluators can actively seek out and ask questions of those with whom they are collaborating about innovative ways to engage interest holders (*36*).

Although discussions regarding how interest holders will make use of the evaluation findings will have occurred in earlier steps, it is important to revisit the planned actions after the evaluation has been implemented because evaluations do not always occur as planned. For example, modifications to data collection procedures (e.g., types of data available, response rates, and sampling) might have changed during implementation and affect how the findings might be best used.

## Applying the Framework: Addressing Evaluation Misconceptions

Three common misconceptions regarding program evaluation are clarified by using this framework. First, the perceived cost of and time required for evaluation can deter their use. The cost of an evaluation depends on the questions asked and the level of precision desired for the answers (*36*,*47*,*48*). A simple, low-cost evaluation can deliver valuable results. However, the expense of an evaluation is relative, and it is important to align the investment in evaluation with program needs. Rather than discounting evaluations as time-consuming and tangential to program operations (e.g., left to the end of a program's project period), the framework encourages conducting evaluations from the beginning that are timed strategically to provide the necessary feedback to guide action. This makes integrating evaluation with program practice possible.

A second misconception centers on the perceived technical demands of designing and conducting an evaluation. Although circumstances exist where controlled environments and elaborate analytic techniques are needed, most public health program evaluations do not require such methods. Instead, the practical approach endorsed by this framework focuses on questions that will improve the program by using context-sensitive methods and analytic techniques that accurately summarize the meaning of quantitative and qualitative information.

Finally, certain program staff might have concerns about evaluation due to perceptions that it is punitive, exclusionary, or adversarial. The framework encourages an evaluation approach that is designed to be helpful and engages all interest holders in a process that welcomes their participation. Penalties to be applied, if any, should not result from discovering negative findings but from failing to use the learning to change for greater effectiveness.

## Conclusion

Program evaluation is an essential activity for any organization interested in understanding and improving their programs and services. The systematic development and implementation of a well-conceived and culturally responsive evaluation can provide insights and recommendations that can only be the result of an evaluation inquiry process. The process of learning and using insights as a collaborative endeavor with interest holders can advance health equity and result in benefits beyond the individual evaluation, such as increased evaluative thinking across organizations, where it becomes part of the culture to ask why something is happening as it is and how to continue to learn and improve.

This framework provides a practical approach to actions, steps, and standards to consider when designing and implementing an evaluation. Because the framework is purposefully general, it provides a guide for designing and conducting specific evaluation projects across many different areas. In addition, using this framework does not preclude using other evaluation approaches, tools, or methods, which can be overlayed and used in conjunction with this framework. Thus, this framework is one of multiple tools that organizations can use to improve their programs and activities.

## Footnotes

* 45 C.F.R. part 46, 21 C.F.R. part 56; 42 U.S.C. Sect. 241(d); 5 U.S.C. Sect. 552a; 44 U.S.C. Sect. 3501 et seq.
